# Convergent evolution of the annual life history syndrome from perennial ancestors

**DOI:** 10.3389/fpls.2022.1048656

**Published:** 2023-01-04

**Authors:** Ane C. Hjertaas, Jill C. Preston, Kent Kainulainen, Aelys M. Humphreys, Siri Fjellheim

**Affiliations:** ^1^ Department of Plant Sciences, Faculty of Biosciences, Norwegian University of Life Sciences, Ås, Norway; ^2^ Department of Plant Biology, The University of Vermont, Burlington, VT, United States; ^3^ Department of Ecology, Environment and Plant Sciences, Stockholm University, Stockholm, Sweden; ^4^ Bolin Centre for Climate Research, Stockholm University, Stockholm, Sweden

**Keywords:** annual, perennial, evolutionary precursors, phylogeny, parallel evolution, convergent evolution, semelparity, iteroparity

## Abstract

Despite most angiosperms being perennial, once-flowering annuals have evolved multiple times independently, making life history traits among the most labile trait syndromes in flowering plants. Much research has focused on discerning the adaptive forces driving the evolution of annual species, and in pinpointing traits that distinguish them from perennials. By contrast, little is known about how ‘annual traits’ evolve, and whether the same traits and genes have evolved in parallel to affect independent origins of the annual syndrome. Here, we review what is known about the distribution of annuals in both phylogenetic and environmental space and assess the evidence for parallel evolution of annuality through similar physiological, developmental, and/or genetic mechanisms. We then use temperate grasses as a case study for modeling the evolution of annuality and suggest future directions for understanding annual-perennial transitions in other groups of plants. Understanding how convergent life history traits evolve can help predict species responses to climate change and allows transfer of knowledge between model and agriculturally important species.

## Introduction

Inquiring into the predictability of evolution, Stephen Jay Gould famously wrote “Replay the tape [of life] a million times … and I doubt anything like *Homo sapiens* would ever evolve again” (Gould, 1989). Although a do-over of Earth’s evolutionary history is not possible to test this hypothesis, the fact that certain traits have evolved multiple times independently – a phenomenon known as convergence – speaks to some level of determinism in the direction of evolution (Mahler et al., 2013; Blount et al., 2018). The nature of this determinism is known to be derived from interplay of similar selective pressures, shared evolutionary history (historical contingency), and the genetic architecture contributing to ancestral phenotypes. Understanding the basis of convergent evolution is critical to accurately predicting organismal responses to climate change. Furthermore, by deciphering the degree to which convergent traits have evolved through the same (“parallel”) mechanisms (**Box 1**) (see Stuart, 2019), we can potentially extrapolate knowledge from model organisms to other close relatives with similar traits and that are agriculturally important or face high extinction risk.

Box 1 **Convergent evolution.** Evolution of a character from one trait/state to another, two or more times independently, regardless of the ancestral trait/state of that character. Also known as homoplasy. See Losos (2011) for a discussion on how this includes early definitions of ‘parallelisms’.**Parallel evolution.** The evolution of a convergent trait involving the same or similar mechanisms. Mechanisms can be defined broadly as sub-traits, developmental pathways, genes, nucleotide changes, epigenetic modifications, etc.**Evolutionary precursor.** An ancestral trait/state that makes convergent evolution of a related trait more likely. Sometimes referred to as a positive constraint.**Phase change.** A time period that marks the resetting of a plant’s physiological state, thus determining the form of its growth and development. The most universal phase change in plants is vegetative to reproductive.**Juvenile-to-adult phase change.** A physiological change during the vegetative period of growth when plants become competent to respond to signals that induce flowering.**Floral competency.** A physiological state attained with age, shortening photoperiods, chilling temperature, and/or their interaction, whereby individual plants can respond to signals, such as lengthening photoperiods and warm temperatures, that induce flowering.

Hundreds of convergent traits have been described for plants, from the relatively simple repeated evolution of bilateral from radially symmetrical flowers (Hileman, 2014), to the more complex multiple origins of C_4_ from C_3_ photosynthesis (Heyduk et al., 2019). In terms of life history traits, transitions between long- and short-day-responsive or high- and low-temperature-induced flowering have occurred repeatedly in angiosperms, as a result of spatio-temporal variations in climate (Preston and Sandve, 2013; Preston and Fjellheim, 2020; Fjellheim et al., 2022; Preston and Fjellheim, 2022). Two additional, intertwined life history traits are generation time and reproductive determinism (Crews and DeHaan, 2015; Salguero-Gomez et al., 2016; Friedman, 2020). At one extreme, such as in the model species *Arabidopsis thaliana* (Brassicaceae), are plants that live less than a year (annuals) and undergo whole-plant senescence upon first reproduction (semelparity/monocarpy). At the other extreme are those that live well over a year (perennials) and reproduce multiple times during their lifetime (iteroparity/polycarpy). Although perenniality and iteroparity usually co-occur, rare but independent origins of perennial, semelparous species have been documented, often associated with habitats that slow growth (e.g., alpine zones) or woody species (e.g., bamboos) with highly synchronous reproduction (Young and Augspurger, 1991; Keeley and Bond, 1999).

**Figure 1:**
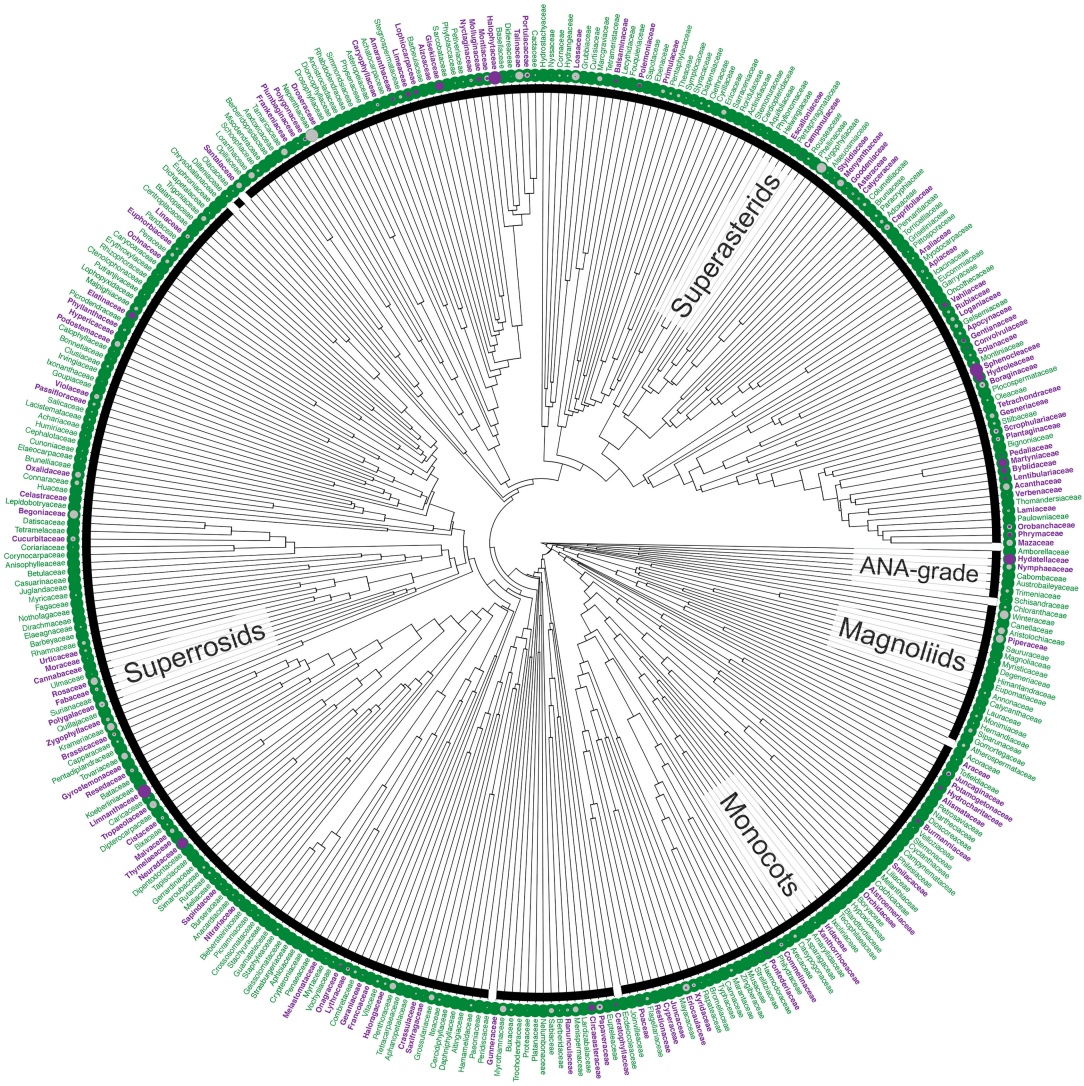
Distribution of annuals among angiosperm families. Families are shown in purple if at least one constituent species is annual; families consisting entirely of perennials are shown in green. Filled circles represent the proportion of the species in a family that is annual (purple) or for which life history data are missing (unknown; grey). The highest numbers of annual species are found in three, large families but the proportion of annuals in these families is not exceptional (Asteraceae, 7%; Fabaceae, 5%), although higher for Poaceae (17%). However, the highest proportions overall are found in small families comprising fewer than 10 species, including the monotypic Halophytaceae (Caryophyllales, Superasterids), Limnanthaceae (Brassicales, Rosids) and Neuradaceae (Malvales, Rosids). Tree based on Magallón et al. (2015); data based on WCSP (2018) and extended upon using the literature and consultation with experts (Supplementary material I, II). The definition of annuals used in this figure includes terrestrial, aquatic and polymorphic species plus those scored as ‘biennials’ in the literature (see Footnote 1).

Despite evidence that generation time and parity can be unlinked (Young and Augspurger, 1991; Salguero-Gomez et al., 2016; Friedman, 2020), a very common transition in plant life history syndromes is from iteroparous perennials (hereafter perennials) to semelparous annuals (hereafter annuals, **Figure 1**). These contrasting strategies are akin to the pace-of-life syndrome in animals (Montiglio et al., 2018), with variation in generation time being specifically determined by predetermined genetic (i.e. age- and senescence-based), rather than broader external (e.g., disease- or accident-based), factors (Nooden and Leopold, 1988; Munne-Bosch, 2008). Differences in both genetically programmed generation time and parity are the product of multiple underlying physiological and developmental traits, and a major unresolved question is the degree to which annual and perennial syndromes have evolved through the deployment of parallel mechanisms.

Here, we outline what is known about the phylogenetic history of annuals and perennials in angiosperms, and briefly review the evidence that annuals have evolved repeatedly in response to seasonal or inter-annual climate extremes. We then examine annuality-perenniality from a developmental perspective to define (1) how genetically programmed generation time and parity develop temporally and spatially within a plant, and (2) what growth and architectural features are correlated with these life history syndromes. Finally, we discuss the extent to which perennial to annual transitions have occurred through parallel changes in flowering time, whole plant physiology, gene expression, and genetic variation; and propose future directions for furthering understanding of the evolutionary basis of life history syndrome convergence.

### Phylogenetic distribution of annuals and perennials in angiosperms

Flowering plants are generally assumed to have originated as perennials, with annuals representing the derived state (Jeffrey, 1916; Ames, 1939). Shifts between life history strategies were long thought to be unidirectional, from perennials to annuals (Stebbins, 1957; Stebbins, 1982), but with increasingly detailed phylogenetic studies, a small number of well-documented cases of annual-to-perennial reversals have come to light. Famous examples are found in the legumes (*Medicago*; Bena et al., 1998, *Lupinus*; Drummond, 2008) and *Castilleja* (Orobanchaceae; Tank and Olmstead, 2008).

Most angiosperms are perennial, with annuals constituting only a small proportion of the species (<10%^1^; **Supplementary Material I, II**; WCSP, 2018). Just three families account for a quarter of all known annuals: the grasses (Poaceae, ca. 1,700 annual species), daisies (Asteraceae, ca. 1,700 annual species) and legumes (Fabaceae, ca. 1,000 annual species). These three families are not each other’s closest relatives but belong to each of the three major clades of angiosperms (Monocots, Asterids and Rosids, respectively; **Figure 1**). In fact, the families that include annuals are distributed across the entire angiosperm phylogeny, including the ANA-grade and the Magnoliids (e.g., in Hydatellaceae and Piperaceae), and, overall, annuals are found in almost a third^1^ of all flowering plant families (**Figure 1**). Thus, the small proportion of species that are annuals have not arisen in a small proportion of lineages; rather, annuals have evolved multiple times independently, in all major clades of angiosperms. Furthermore, the distribution of annuals among clades is not random, but phylogenetically structured (Supplementary material III), suggesting a strong evolutionary signal to the factors underlying the convergent evolution of annuals. Understanding the nature of these underlying factors, whether environmental, genetic, and/or developmental, is a matter of broad appeal.

### Environmental factors driving convergent evolution of annual traits

**Figure 2:**
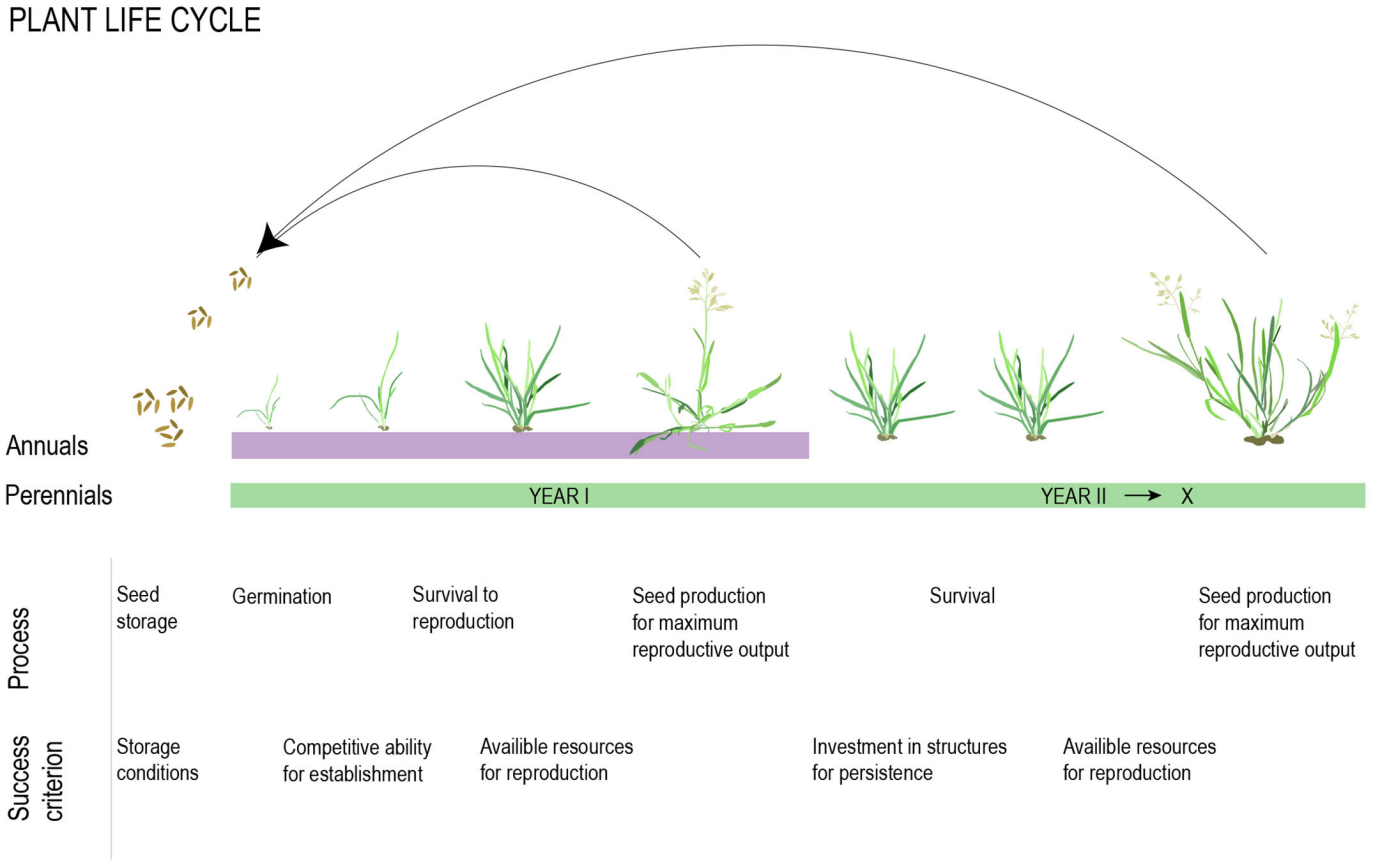
Developmental and physiological processes influencing reproductive output in annual and perennial taxa. The total outcome is determined by resources available for allocation to these processes and the trade-offs between them.

Selection for one life history strategy over another is likely to depend on their respective prospects for maximizing lifetime seed set, which is minimally determined by survival to reproduction, average viable seed yield per reproductive season, and number of reproductive seasons (**Figure 2**, Stearns, 1992). Given no evolutionary constraints, the outcome of selection will depend on the ratio between adult and juvenile survival, where annuals will be favored if juvenile survival is high relative to adult survival and establishment (**Figure 2**, Stearns, 1992). This balance is largely determined by environmental factors, and rapid life cycles are, in general, favored when environmental variation across seasons is high, but year-to-year variation is low (Charnov and Schaffer, 1973; Stearns, 1992; Franco and Silvertown, 1996; Monroe et al., 2019). In this section, we briefly discuss what we know about the role of environmental factors in shaping life history variation. We also discuss the less well-documented patterns of phylogenetic relatedness within a community in relation to community-level selection for annuals versus perennials.

Several studies suggest that annual plants have a selective advantage over perennials in seasonally dry environments. Part of this advantage comes from the fact that a successful annual seedbank depends on dry storage conditions and that annuals have an enhanced ability to outcompete already established perennials under drought conditions during germination and seedling growth (Corbin and D'Antonio, 2004; Monroe et al., 2019). Annual species likely also become more competitive under seasonally stressful and high disturbance conditions – such as drought, heat, flooding, grazing, and erosion – which, unlike perennials that need to invest in persistence, annuals can escape as seed (Whyte, 1977; Pettit et al., 1995; Tofts, 2004; Evans et al., 2005; Kadereit et al., 2006; Cruz-Mazo et al., 2009; Nunes et al., 2019; Massante et al., 2021). Although most studies indicate that annuals are frequent in areas with predictable drought, we are still in need of large empirical studies to test the generality of these results. Interestingly, high-latitude winters, which are among the most stressful seasonal environments for plants, do not appear to have selected for increased annuality (Lindberg et al., 2020; Massante et al., 2021). One explanation for this is that lower growth rates at cold temperatures can limit a plant’s ability to complete its life cycle during the short growing season (Körner and Larcher, 1988; Ogburn and Edwards, 2015). A further explanation might be that conditions for seed banks are suboptimal at high latitudes, where soils can be relatively warm and moist under the snowpack. The latter hypothesis remains to be investigated.

Annual species are repeatedly found in similar types of environments, indicating that the same selective pressures are most likely at play in most perennial to annual transitions. However, as most studies do not account for phylogeny, it is not known if transitions happen through independent or parallel mechanisms, and/or if origins of annuality are overestimated by including annual species that evolved from a common annual ancestor. In studies of species community assemblages in specific environments, it has been suggested that phylogenetic over-dispersion of species (i.e., species are less related than expected by chance) is the result of independent convergent evolution, whereas constraints and/or conservation of functional traits through parallel mechanisms would lead to non-random clustering of annual species (Cavender-Bares et al., 2009). Recently, some studies of community assemblages in arid environments have taken phylogeny into account (García-Camacho et al., 2017; Massante et al., 2021). Massante et al. (2021) studied species richness and relatedness across a climate gradient of the arid Mediterranean and found lower species richness in more arid regions for perennial, but not annual, species. Furthermore, they found that perennial species showed overdispersion across an aridity gradient in the Mediterranean, indicating independently converged traits, whereas annuals were phylogenetically clustered at different phylogenetic scales in areas of medium to high aridity, evidencing single origins of the annual syndrome within distinct regions. In contrast to this result, a study by García-Camacho et al. (2017) showed that over-dispersion of annual species in dry areas was the result of independent convergent evolution of drought resistance strategies. It must be noted that other processes besides adaptation, such as environmental filtering and dispersal, could contribute to strong phylogenetic structure in arid communities, and the underlying factors shaping the community assemblages must thus be investigated specifically.

The fact that annual and perennial species are often found in sympatry also suggests that similar environments can select for different life history strategies, and/or that not all plants are able to optimize their strategies due to temporal, historical, or developmental constraints and trade-offs. In other words, environmental factors cannot be the whole explanation for the convergent evolution of annuality. Exaptations, defined as the cooption of an existing trait for a novel function, might also create seemingly convergent phenotypes (Losos, 2011). For example, it is possible that annual species adapted to seasonal drought disperse more easily into novel unstable environments than their perennial counterparts.

### Developmental and physiological correlates of life history trait variation in model taxa

Annual and perennial life histories are not particularly well-defined or discrete traits, but rather compound quantitative traits that together contribute to trade-offs between survival to reproduction, seed production, and continued vegetative growth following senescence. The widespread convergence of annual syndromes (**Figure 1**) not only offers a means to investigate which traits are required for a particular life history strategy, but also to determine the extent to which parallel mechanisms have been deployed in the evolution of individual characters constituting these syndromes. In this section, we begin addressing these issues by describing what is known about the physiological and developmental correlates of annuality versus perenniality across flowering plants. We then suggest future avenues for comparing these quantitative traits across phylogenetically diverse clades with variation in life history syndromes.

#### Variation in growth traits

**Figure 3:**
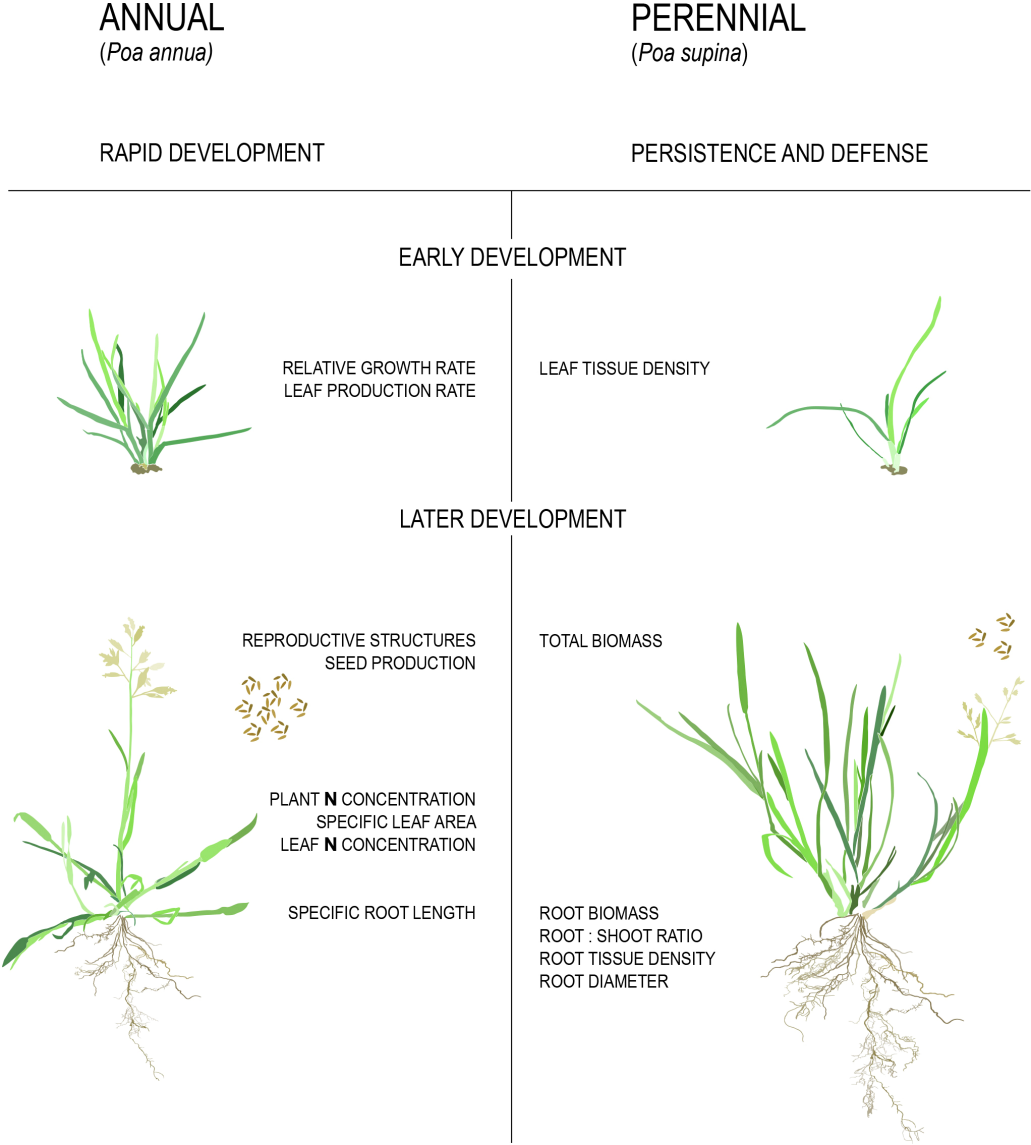
Physiological differences between annual and perennial plants. Traits listed in the figure are higher/larger in the respective strategies.

It has been shown repeatedly that annual plants maximize their reproductive effort through high relative growth rates (RGRs), large leaf areas, large allocation of resources to reproductive structures, and high biomass production and specific root lengths (**Figure 3**, Grime and Hunt, 1975; Bazzaz et al., 1987; De Souza and Da Silva, 1987; Garnier, 1992; Roumet et al., 2006; Atkinson et al., 2016; Poorter et al., 2009). By contrast, perennials maximize persistence, defense, and stress tolerance through high above- and below-ground tissue density and allocation of a higher proportion of biomass to roots (**Figure 3**, Grime and Hunt, 1975; Bazzaz et al., 1987; De Souza and Da Silva, 1987; Garnier, 1992; Roumet et al., 2006; Atkinson et al., 2016). For example, in a set of studies on congeneric annual and perennial species pairs of grasses it was found that similar physiological traits distinguished the two life history syndromes (Garnier, 1992; Garnier and Laurent, 1994; Garnier and Vancaeyzeele, 1994; Garnier et al., 1997). It should be noted that these studies failed to correct for phylogenetic relatedness, potentially inflating the replicates available for comparison. Furthermore, it is unclear what the ancestral state for each trait was, and whether the complex of ‘annual’ or ‘perennial’ traits evolved in a similar order.

A meta-analysis that did account for phylogeny considered 3,000 congeneric annual and perennial species pairs from across the angiosperms (Vico et al., 2016). As expected, perennials had a higher fraction of root mass to total biomass, root:shoot ratio and dry biomass of both roots and shoots relative to annuals whereas annual species had a higher fraction of seed mass to total biomass, CO2 assimilation rate per unit leaf area, specific leaf area, relative growth rate, and N content in both leaves and the whole plant relative to perennials. These data convincingly demonstrate several traits that are part of the life history syndromes through convergent evolution. However, it should be noted that the difference in single seed mass and seed dry biomass between annual and perennial species disappeared when accounting for cultivation status (Vico et al., 2016). This suggests that at least some of the traits seemingly associated with annuals might really be correlated with domesticated crops, such as rice (*Oryza sativa*), maize (*Zea mays*), wheat (*Triticum* sp.) and sorghum (*Sorghum bicolor*).

Indeed, in a study of wild and cultivated bean (*Phaseolus*), there were only slight differences in a range of traits between annual and perennial wild species, whereas there were large differences in trait values between cultivated annual and perennial species (Herron et al., 2020). Igea et al. (2017) also performed a major study of seed mass evolution across angiosperms and found no differences between annuals and perennials. However, Humphreys et al. (2011) analyzed a small clade of Southern Hemisphere grasses and did find differences in seed characteristics between annual and perennial species, such as presence of awns, hairs, and overall size. The latter authors speculated that differences were related to the different burial mechanisms employed by annual and perennial species, which in turn relate to their different requirements for a seedbank.

#### Variation in the extent and timing of senescence, and its role in parity

**Figure 4:**
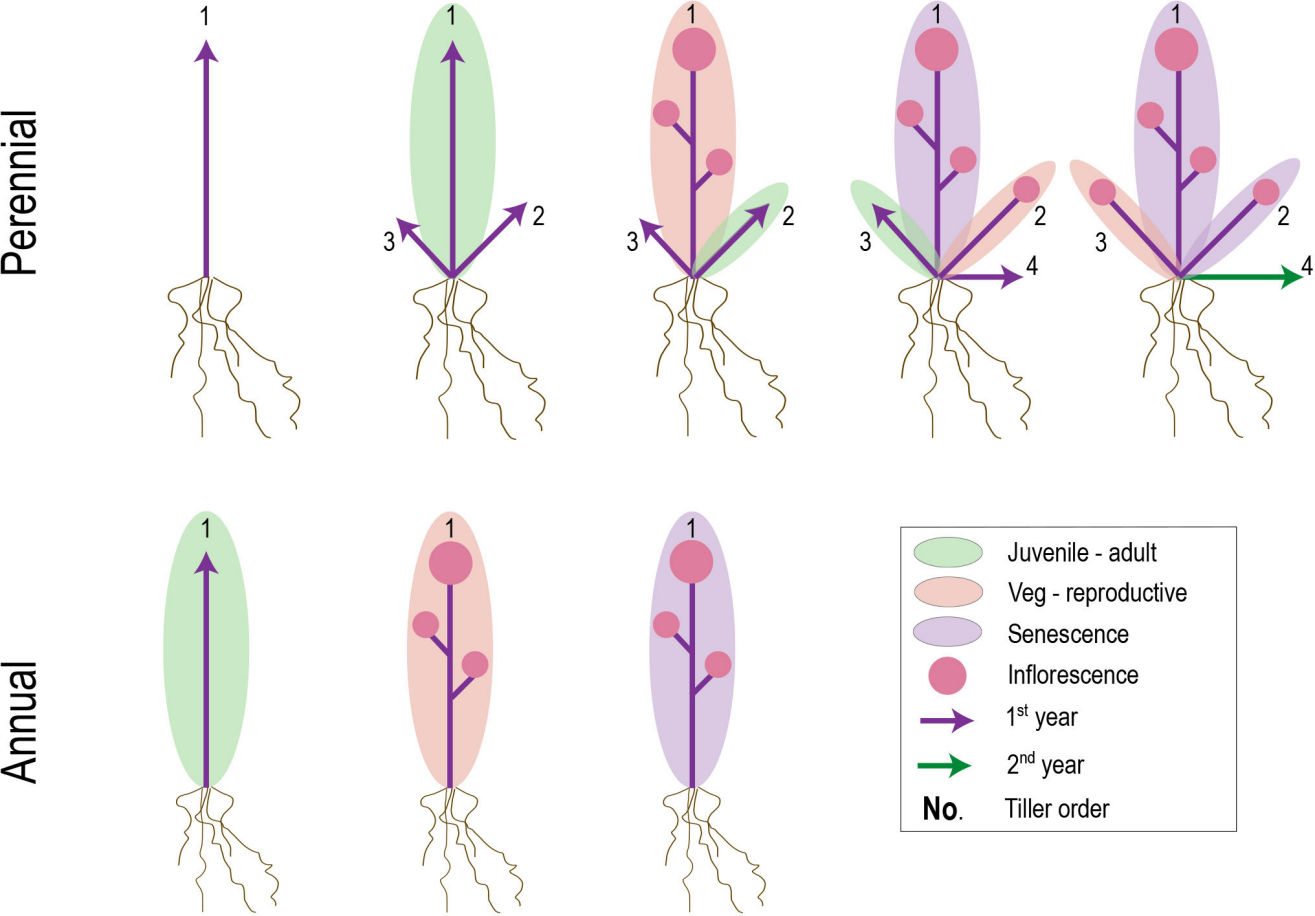
Hypothetical figure suggesting common mechanisms underlying transitions between perennial and annual life histories. Evidence from at least some plant groups with mixed perennial and annual habits suggests heterochronic shifts in juvenile-adult (green ellipse) and/or vegetative-reproductive (yellow ellipse) phase transitions. Relatively later transitions in perennial plants, combined with a potentially larger root to shoot system, might allow increased shoot meristem development and/or outgrowth prior to first flowering. If phase transitions and senescence occur successively on individual branches (labeled 1-4), late-developing branches in perennials that are juvenile upon the first waves of senescence might be preserved in that state until the next growing season.

Individual lifespan in plants is determined by a combination of accidents, aging, and global senescence (Nooden and Leopold, 1988). However, whereas accidents and aging are likely to define population-level variation in generation times of perennials, the occurrence and/or timing of genetically programmed global senescence is the key determinant of lifespan differences *between* annuals and perennials (Munne-Bosch, 2008). Senescence can be defined as the programmed cell death (PCD) of whole organs and is employed universally across plants to maintain nutrient balance and provide defense against damage (Lim et al., 2007), often in association with specific seasons and life history stages (Estiarte and Peñuelas, 2015). In creeping perennials, post-reproductive vegetative offspring (ramets or plantlets) often undergo large-scale senescence, but premature mortality is avoided at the colony (genet or whole plant) level through the outgrowth of axillary meristems that develop beyond the wave of PCD (Thomas et al., 2000) (**Figure 4**). Shrubs and trees show similar pulses of large-scale senescence, but in contrast to horizontally spreading perennials, regions of dead biomass are maintained and recruited into structural support and nutrient transport (Thomas et al., 2000).

In the case of annuals, post-reproductive senescence resembles that of perennial ramets. However, a critical difference is that no meristems escape from the leading edge of PCD, leading to semelparity (**Figure 4**). The signal for global senescence comes from hormone signaling by fruits and seeds (Malik and Berrie, 1975; Lim et al., 2007; Ware et al., 2020; Miryeganeh, 2021), but what defines the boundary around the senescence zone has only recently started to be elucidated. Research in *A. thaliana* suggests that senescence manifests locally within each inflorescence and occurs only when (1) the branch is competent to respond to senescence signals and (2) an auxin signal comes from fruit proximal to the branch apex (Ware et al., 2020). Given this localized senescence model, the question remains as to why the underlying vegetative leaves undergo PCD in concert with the inflorescences.

One possible explanation for whole-plant senescence in annuals is that, unlike the inflorescences, senescence in vegetative leaves is triggered by global source-sink dynamics, in which case annuals that dedicate a large proportion of meristems/biomass to fruit production will undergo whole-plant senescence at first flowering. Alternatively, leaves near senescing inflorescences might be more likely to receive senescence signals than those farther away, such as on dedicated vegetative branches. Indeed, in the agricultural practice of ratooning, annual monocots such as rice, pineapple, and sugarcane can be forced to produce a second annual crop from young axillary meristems when the older reproductive branches are removed (Plucknett et al., 1970). Whether this can be interpreted as a change in parity or simply a delay in reproductive output is a matter of debate. Nonetheless, removal of the senescence signal appears to block immediate senescence of the remaining meristems.

Finally, spatio-temporal differences in senescence between annuals and perennials might be linked to variation in developmental age that provides competency to respond to PCD signals (Balanzá et al., 2018). In hybrids between temperate annual and perennial grasses, longevity appears to be a quantitative trait, with perennial genome dosage affecting the number of subsequent reproductive bouts (von Bothmer et al., 1983; Thomas, 1995; Jones et al., 1999). Hybrids thus represent an important resource for analyzing architectural and source-sink differences associated with the timing of whole-plant senescence, as do comparative analyses in annual-perennial pairs and classical experiments manipulating fruit load and subsequent hormone signaling (Ware et al., 2020).

#### Variation in the time to first flowering

**Figure 5:**
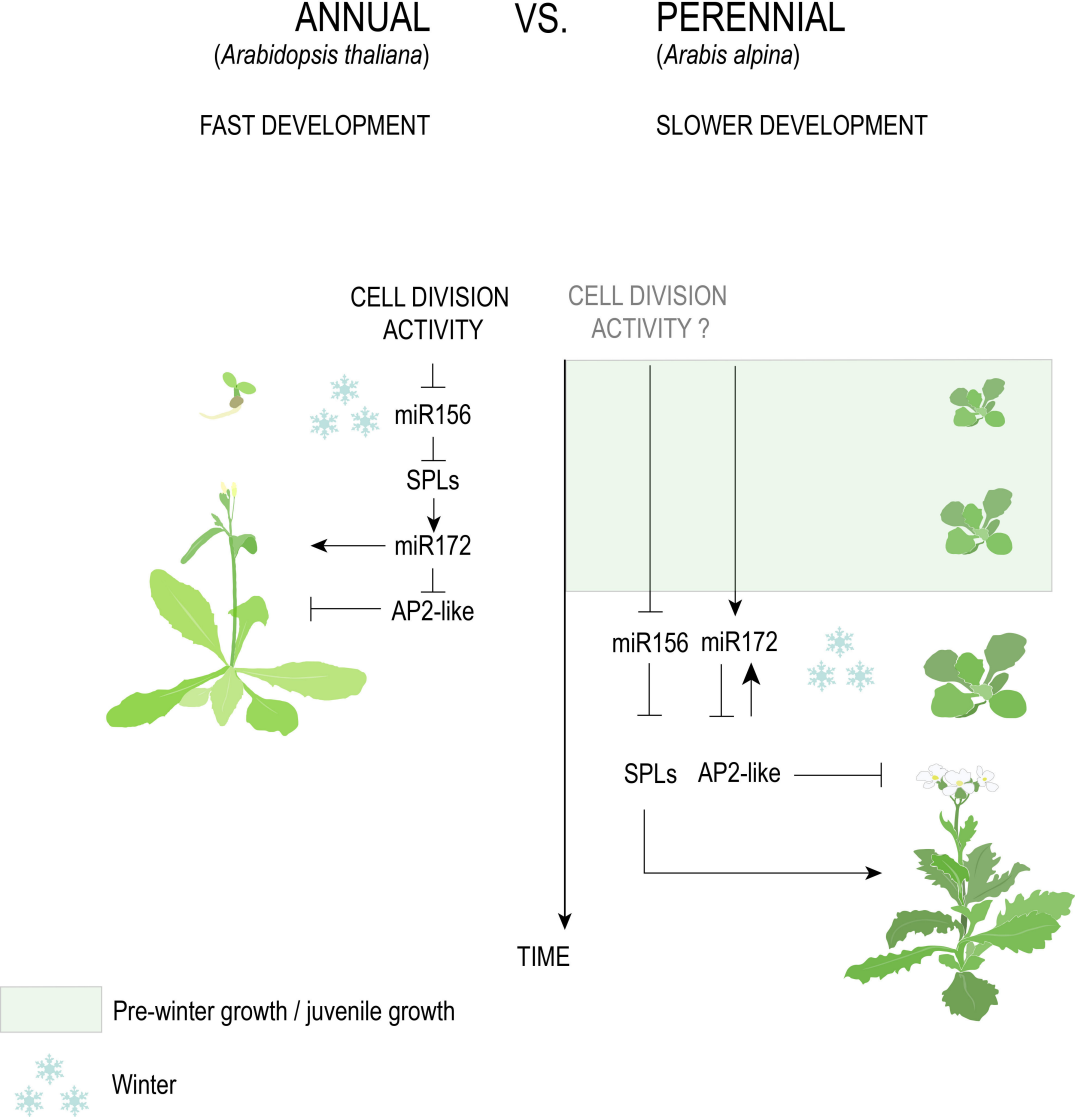
The genetic pathway of age in Brassicaceae. The antagonistic age-dependent relationship between miR156 and miR172 negatively regulates floral activators (SPLs) and floral repressors (AP2-like), respectively. A fast decline of miR156, triggered by cell division activity, in *A. thaliana* versus *A. alpina*, explains the former’s response to vernalization at a very early age and why the latter requires an extended period of growth before cold exposure.

In addition to the occurrence and/or timing of global senescence, a potentially important trait distinguishing annuals from perennials is the length of time to first flowering (**Figure 5**; Bergonzi and Albani, 2011). Flowering time is controlled by a combination of internal and external signaling pathways that converge at the shoot apical meristem (SAM) to affect developmental phase change. The ability to flower in response to inductive conditions (e.g., warm lengthening days for temperate taxa) is often predicated on an individual achieving floral competency (**Box 1**) which in turn can be attained through increasing development age, shortening photoperiods, cooling (vernalizing) temperatures, and/or their interaction (Poethig, 2003). For example, unlike their annual relative *A. thaliana*, Brassicaceae perennials such as *Arabis alpina* and *Cardamine flexuosa* show increased sensitivity to vernalization as plants age (Bergonzi et al., 2013; Zhou et al., 2013). As a result, individuals of these species are often able to achieve floral competency only after two winters. Furthermore, the length of time it takes to saturate the vernalization response can vary considerably among populations and species. To test the hypothesis that time to first flowering is accelerated in annuals relative to perennials, and that this is due to stronger age-dependent vernalization sensitivity and/or increased saturation time (**Figure 5**), we advocate for systematic experimentation across diverse annual-perennial species pairs. This can be done by measuring the time from germination to inflorescence production following different periods of vernalization and giving fixed vernalization periods at variable times post-germination.

In addition to flowering time, developmental age in some species influences the timing and morphology of traits such as spring leaf-out and specific leaf morphology. In *A. thaliana*, for example, so-called juvenile stage plants have round hairy leaves, enhanced rooting ability, and are often incompetent to flower; whereas adult plants have narrow leaves, reduced rooting ability, and are often competent to flower (**Box 1**; Evans and Poethig, 1995; Telfer et al., 1997). The length of the juvenile phase has been investigated for several taxa, and ranges from just a few days in *A. thaliana* to several years, as in the case of olive (*Olea europaea*) (Zimmerman, 1972; Sgamma et al., 2014). Despite this, few studies have explicitly compared the length of the juvenile phase between closely related annual-perennial pairs to test the hypothesis that adult phase onset is delayed in perennials.

### Molecular basis for convergence in life history trait variation

Most comparative genetics on annuals versus perennials have focused on differences in the genes involved in the timing and complexity of phase transitions, whereas the highly polygenic basis of growth trait variation has only begun to be deciphered (Alonso-Blanco et al., 2009). In this section we briefly describe some of the known genetic pathways involved in growth, phase change, and senescence, and discuss how they might have been modified to affect life history transitions. We also recommend avenues for future research, particularly focusing on studies that will be required to test for parallel evolution.

#### Growth trait variation

We have little knowledge about the genomic architecture of above and belowground growth traits that partially explain differences between annual and perennial species (Vico et al., 2016). Previous work primarily focused on candidate genes known to be involved in cell division or elongation in model species, or analyses comparing gene content and substitution ratios to find evidence of adaptive evolution in annual versus perennial species. The latter approach, for example, found five candidate gene families - kinases, oxidoreductases, lactoylglutathione lyases, F-box proteins and zinc-fingers – for growth differences between annual/perennial sister species in Brassicaceae, many of which await functional validation (Heidel et al., 2016).

An interesting group of genes that have started to be investigated relative to aboveground growth are plant cyclins that control commitment to cell division. Overexpression of one of these proteins in tobacco (*Nicotiana tabacum*) resulted in more aboveground mass and faster flowering relative to non-transgenic plants (Cockcroft et al., 2000). In the *Festuca-Lolium* species complex, cyclins have been identified as candidates for growth habit differences based on differential expression between annual and perennial members (Czaban et al., 2015).

Ligases have also been explored in relation to regulation of above- to below-ground resource allocation, for instance the E3 ubiquitin transferase/ligase *PUB4* in *A. thaliana*. Single nucleotide polymorphisms (SNPs) in this gene are associated with both allometric scaling and hot temperatures, making it a strong candidate for a trait related to life history (Vasseur et al., 2018). Interestingly, E3 ubiquitin ligases have also been identified as candidates for involvement in evolution of life history in annual-perennial, but not in perennial-perennial species comparisons of the *Festuca-Lolium* complex (Pooideae) (Czaban et al., 2015). If *PUB4* is found to be involved in growth differences between annual-perennial Brassicaceae, this result would suggest parallel recruitment of paralogous ligases for allometric scaling in the convergent origin of annuality in Brassicaceae versus grasses.

#### Variation in discrete morphological traits

Beyond whole plant growth, many perennial species can be differentiated from their annual counterparts by the presence of continuously growing vegetative structures, such as rhizomes, stolons, and tillers, which contribute to sustain perennial plants during unfavorable conditions. In rice and sorghum (*Sorghum* sp.), the loss of rhizome production has been mapped to different mutations in *RHZ2* and *RHZ3*, again suggesting parallel evolution of this trait (Hu et al., 2003). In contrast, introgression of the short arm of chromosome four from perennial *Thinopyrum elongatum* to its close bread wheat relative results in regrowth from tillers (Lammer et al., 2004), and this has been mapped to variation at *POST SEXUAL CYCLE REGROWTH 1* (*PSCR1*) (Abbasi et al., 2020). *PSCR1* is the likely orthologue of *TEOSINTE BRANCHED 1* (*TB1*) that together with *GRASSY TILLERS 1* (*GT1*) suppresses tiller outgrowth in maize (Doebley et al., 1995; Whipple et al., 2011; Wills et al., 2013; Dong et al., 2019; Abbasi et al., 2020). *TB1* and *GT1* have been implicated in tiller suppression in the derived annual species wheat, rice, and green foxtail (*Setaria viridis*) (Hu et al., 2018; Dong et al., 2019). The gene *TILLER NUMBER 1* (*TIN1*) controls tiller number in maize by repressing *GT1* (Zhang et al., 2019; Swentowsky et al., 2021) Furthermore, perennial regrowth in the maize relative *Zea diploperennis* has been linked to the QTLs *REG1, REG2* (Ma et al., 2019), and *REG3* (Swentowsky et al., 2021) that putatively encode from *GT1* and its upstream negative regulator *TIN1* (Swentowsky et al., 2021). Future studies comparing *TB1*, *TIN1*, and *GT1* in different annual-perennial pairs will be required to directly test the hypothesis that mutations in these genes were responsible for convergent evolution of annual traits across grasses, particularly given that they are candidates for tillering in some annual grasses and regrowth in others (Hu et al., 2018; Dong et al., 2019; Swentowsky et al., 2020).

#### Extent and timing of senescence

The onset and extent of senescence clearly have major impacts on plant lifespan and life strategy. However, finding a genetic basis for variation in senescence has been hampered by a general focus on this process in annual species, and the complex nature of senescence signaling that is affected by the overall architecture of a plant and its resource availability (**Figure 4**) (Wu et al., 2012). Thomas (2013) ponders the idea that “the difference between annuality and perenniality is essentially quantitative, the consequence of genetically determined variations in the balance between rates inward [i.e., organogenesis and recruitment] and rates outward [i.e., exit through death]”. Assuming a conserved and highly coordinated leaf senescence program, genes that affect growth rates (see previous section) might be pursued as good candidates for explaining the time at which senescence becomes global. Likely other explanators would be genes involved in differential competency to respond to senescence signals, such as those involved in establishing developmental age (see next section), and modifiers of the senescence pathways themselves.

From a genetically programmed perspective, all leaves in *A. thaliana* have the same lifespan, which can be defined as short relative to the slowing growth rate reached with the onset of reproduction. The age at which these leaves become susceptible to senescence signals is defined by several antagonistic proteins, including the senescence repressor ONSET OF LEAF DEATH (OLD) and the senescence activators OESARA1 (ORE1) and ETHYLENE INSENSITIVE3 (EIN3) (Qiu et al., 2015). Leaf senescence signals, such as the hormones ethylene and jasmonic acid, appear to be regulated by both developmental age and stress. For example, increased drought in the US Corn Belt led to premature leaf senescence of maize, which in turn decreased yield as a result of reduced nitrogen and sugar sources (Wu et al., 2012). In rice and other annual plants, variation at the *STAYGREEN* (*SGR*) locus that encodes a chlorophyll-degrading Mg++-dechelatase affects the age of whole plant senescence, thus modifying lifespan (Shin et al., 2020). Although we know of no study that explicitly compares the action of OLD, ORE1, EIN3, and SGR in annual-perennial pairs, partial silencing of *SGR* in perennial ryegrass (*Lolium perenne*) increases forage quality without any obvious effects on flowering time (Xu et al., 2019). These data suggest that differences in the regulation and/or biochemical function of proteins such as SGR could, in principle, underlie differences in the maintenance of energy sources required for continued vegetative growth at reproduction.

Despite limited work in perennials, data suggest general conservation of the senescence process across life strategies that involves macromolecule degradation and recycling, nutrient remobilization, detoxification, and finally cell death (Lim et al., 2007; Minina et al., 2013). Several senescence-associated genes (SAGs) are responsible for these changes, but it remains unclear how conserved the developmental function of these genes is between annual-perennial pairs. In the perennial shrub *Catharanthus roseus*, lifespan is extended by *LEAFLESS INFLORESCENCE* (*LLI*) and shortened by *EVERGREEN DWARF* (*EGD*). However, the fact that *LLI* mutants do not become monocarpic cautions against these genes being strong candidates for transitions to annuality (Kumari et al., 2010). Moving forward, a particularly intriguing system for understanding variation in senescence-based lifespan is the perennial rice hybrid *O. sativa* x *O. longistaminata*. Unlike annual *O. sativa*, this hybrid can produce abundant twice-yearly harvests for up to four years, resulting in significantly easier, cheaper, and more sustainable farming (Zhang et al., 2022).

#### Age pathway

The length of the juvenile stage is one character hypothesized to distinguish annuals from perennials that are derived from a recent common ancestor. A highly conserved system for determining the juvenile-to-adult transition involves two microRNAs: miR156, which inhibits the expression of *SQUAMOSA PROMOTOR-BINDING PROTEIN-LIKE* (*SBP/SPL*) genes involved in accelerating adult phase change and flowering, and its antagonist miR172 that inhibits the expression of *APETALA2* (*AP2*)-like genes involved in repressing flowering (**Figure 5**, Aukerman and Sakai, 2003; Axtell and Bowman, 2008; Teotia and Tang, 2014; Wang and Wang, 2015; Ma et al., 2020). As plants age and stored photosynthate increases, miR156 levels decrease, resulting in the de-repression of SPL genes (Yu et al., 2015; Hyun et al., 2017). At least in *A. thaliana*, de-repression of SPLs causes the upregulation of miR172, together marking the transition to adult growth and eventual flower production (Wu et al., 2009).

Despite strong evidence that the miR156/SPL and miR172/AP2 modules are conserved across angiosperms regardless of life history, differential regulation of these modules in annuals versus perennials has been posited. In *A. thaliana*, the activity of Polycomb Repressive Complex 1 (PRC1) and 2 (PRC2), along with cell division rate in the apical meristem, promotes the temporal decline of miR156, demonstrating that developmental, rather than absolute, age controls the juvenile-to-adult transition (Xu et al., 2016a; Xu et al., 2018; Cheng et al., 2021). Emerging evidence of a faster decline in miR156 levels in annual versus perennial species (Park et al., 2017) could therefore suggest that higher growth rates allow annuals to reach a certain developmental age faster (**Table 1**). For temperate species that require vernalization, this would predict that annual species acquire the competency to respond to winter temperatures faster than perennials.

**Table 1:** The antagonistic age-dependent relationship between miR156 and miR172 in annuals (A) and perennials (P).

Species		Habit	MIR156 high/low(er)	MIR172 low/high(er)	Reference
*Zea mays*	Poaceae	A	Day 7/day 14	(Week 4)	Chuck et al., 2007
*Hordeum vulgare*	Poaceae	A	Day 11	Day 13	Tripathi et al., 2018
*Triticum aestivum*	Poaceae	A	Leaf 1-3/leaf 5-7	Leaf 1-5/leaf 7	Debernardi et al., 2022
*Sorghum bicolor*	Poaceae	A	Leaf 1-4/leaf 5-6	Leaf 1-3/leaf 4-6	Hashimoto et al., 2019
*Oryza sativa*	Poaceae	A	Leaf 1-2/leaf 2-3	Leaf 1-2/leaf 4-6	Tanaka, 2012
*Panicum virgatum*	Poaceae	P	Seedling/adult		Matts et al., 2010
*Arabidopsis thaliana*	Brassicaceae	A	Day 12/day 19	Day 12/day 19	Wu et al., 2009
*Cardamine flexuosa*	Brassicaceae	P	Week 1-3/week 4	Week 1-2/week 3-4	Zhou et al., 2013
*Arabis alpina*	Brassicaceae	P	Week 1-5/week 6	Week 1-5/week 6-8	Bergonzi et al., 2013; Zhou et al., 2020
*Soybean*	Fabaceae	A	Leaf 1-2/leaf 3-4	Leaf 1-4/leaf 5-6	Yoshikawa et al., 2013
*Acacia confusa*	Fabaceae	P	Juvenile leaf/adult leaf	Juvenile leaf/adult leaf	Wang et al., 2011
*Acacia colei*	Fabaceae	P	Juvenile leaf/adult leaf	Juvenile leaf/adult leaf	Wang et al., 2011
*Nicotiana tabacum*	Solanaceae	P	Day 1-20/day 25	Day 1-20/day 25	Feng et al., 2016
*Passiflora edulis*	Passifloraceae	P	Leaf 3/leaf 6	Leaf 3/leaf 10	Silva et al., 2019
*Fragaria × ananassa*	Rosaceae	P	Month 1-2/month 2	Month 1-2/month 2	Li et al., 2012
*Malus*	Rosaceae	P	Node 1-40/node 80	Node 1-40^/^node 80	Du et al., 2015
*Populus x canadensis*	Salicaceae	P	1 month/1 year	1 month/Year 1	Wang et al., 2011
*Eucalyptus globulus*	Myrtaceae	P	Juvenile leaf/adult leaf	Juvenile leaf/adult leaf	Wang et al., 2011
*Hedera helix*	Araliaceae	P	Juvenile leaf/adult leaf	Juvenile leaf/adult leaf	Wang et al., 2011
*Quercus acutissima*	Fagaceae	P	Juvenile leaf/adult leaf	Juvenile leaf/adult leaf	Wang et al., 2011
*Paeonia ostii*	Paeoniaceae	P	Year 1-2/year 3	Year 1-2/year 3	Han et al., 2021
*Persea americana*	Lauraceae	P	Month 1-year 1.5/year 10	Month 1-3/year 1.5-10	Ahsan et al., 2019
*Macadamia integrifolia*	Proteaceae	P	Month 1-4/year 1-5	Unchanged	Ahsan et al., 2019
*Mangifera indica*	Anacardiaceae	P	Month 1-6/year 10	Unchanged	Ahsan et al., 2019

Annuals such as *A. thaliana*, bread wheat (Hoogendoorn, 1984), barley (Sasani et al., 2009), and *B. distachyon* (Schwartz et al., 2010) respond to vernalization immediately after sowing and show relatively rapid declines in miR156 transcription, with expression in the former species being synchronized in axillary branches irrespective of chronological age (Park et al., 2017). By contrast, the perennials *Arabis alpina* and *Cardamine flexuosa* require five weeks of growth before cold exposure (Bergonzi et al., 2013; Zhou et al., 2013), the former of which shows a slow reduction in miR156 transcription that is autonomously regulated in different meristems (Park et al., 2017; Ponraj and Theres, 2020). A recent study also found a delayed accumulation of miR172 in *A. alpina* relative to *A. thaliana* that negatively corresponds to miR156 levels prior to vernalization (Zhou et al., 2020). This is at odds with previous results that suggested a requirement for vernalization to increase miR172 in the Brassicaceae perennial (Bergonzi et al., 2013).

While little is known about the regulation of miR156 in species outside of *A. thaliana*, a study into rice implicates PRC1 and 2 in both conserved and divergent roles of phase transitions. Mutants of the putative *LIKE HETEROCHROMATIN PROTEIN 1* (*LHP1*), a PRC1/PRC2 accessory protein, in rice revealed an extended juvenile phase with delayed flowering (Cui et al., 2020). Although the *A. thaliana BMI1*-*prc1* loss-of-function showed a similar phenotype, *A. thaliana lhp1* mutants actually have a shortened juvenile phase and flower earlier than wild-type plants (Bechtold et al., 1993; Gaudin et al., 2001; Exner et al., 2009; Pico et al., 2015). In addition, several other agents involved in modulating miR156 expression have been identified, such as the chromatin regulators PICKLE (Xu et al., 2016a), BRAHMA *(* Xu et al., 2016b), HTA9/11 (Choi et al., 2016), ARP6 (Xu et al., 2018) and MSI1 (Kumar et al., 2020), the transcription factors AGAMOUS-like 15 and 18 (Serivichyaswat et al., 2015), MYB33 (Guo et al., 2017; Guo et al., 2021) and members of the NUCLEAR FACTOR Y family (Wei et al., 2017; Zhao et al., 2020) as well as *VAL* genes (Fouracre et al., 2021). Whether these or other signals are involved in differentially regulating the temporal expression of miR156 to orchestrate delayed phase transitions in annuals versus perennials is unknown. Nonetheless, direct manipulation of miR156 levels under the gibberellin 3 oxidase 2 (GA3ox2) promoter increased branching and overall grain yield in both seasonal and ratooning rice, suggesting a strong link between developmental age, architecture, and productivity (Liu et al., 2019).

#### Autonomous and vernalization pathways

Beyond the age pathway, the juvenile-to-adult transition in Brassicaceae is controlled by down-regulation of the flowering repressor FLOWERING LOCUS C (FLC) that is part of both the autonomous and vernalization pathways (Michaels and Amasino, 1999; Whittaker and Dean, 2017; Kiefer et al., 2017; Soppe et al., 2021). In winter annual accessions of *A. thaliana*, FLC is epigenetically silenced when the plant experiences prolonged cold and remain in a stable silenced state until embryogenesis, where it is reset to ensure a vernalization response in the following generation (Sheldon et al., 2008; Crevillén et al., 2014). Crucially, the stable silenced state occurs at the whole plant level allowing for activation of inflorescences in all meristems. In perennial relatives of *A. thaliana*, FLC orthologs exert similar yet distinct functions. Studies into the perennial relative *A. alpina* have implicated the *FLC* ortholog *PERPETUAL FLOWERING 1 (PEP1)* in contributing to several aspects of the perennial life history (Wang et al., 2009; Albani et al., 2012; Hyun et al., 2019). In the vernalization-requiring *A. alpina* accession “Pajares”, *PEP1* is stably silenced after 18 weeks of cold, similar to *FLC* in *A. thaliana* accessions that require longer periods of cold to flower (Lazaro et al., 2018; Nishio et al., 2020). By contrast, *A. alpina PEP1* is only stably silenced in older shoots (Bergonzi et al., 2013; Park et al., 2017; Lazaro et al., 2018). Given that miR156 determines the age at which meristems become responsive to vernalization, it has been hypothesized that differential spatial patterns of *FLC*/*PEP1* silencing are controlled by variation in the age pathway and/or upstream elements in the autonomous pathway (Bergonzi et al., 2013). The functionality of *PEP1* alleles does not affect longevity and parity per se (Albani et al., 2012) but might contribute to the success of the perennial syndrome in seasonally cold climates (Hughes et al., 2019).

### Exaptations - evolutionary precursor traits

Despite fundamental differences in growth strategies between annual and perennial species, the evolutionary distance between them can be minimal (van Kleunen, 2007), indicating that small differences in genetic makeup differentiate their growth habits. Nonetheless, the majority (two-thirds) of plant families do not contain annual species at all, and the distribution of annual species across the angiosperm phylogeny is not even (**Figure 1**). A key question then, is to what extent the non-random phylogenetic distribution of annual species is due to an absence of selective pressures versus a predisposition to evolve traits associated with the annual syndrome.

**Figure 6:**
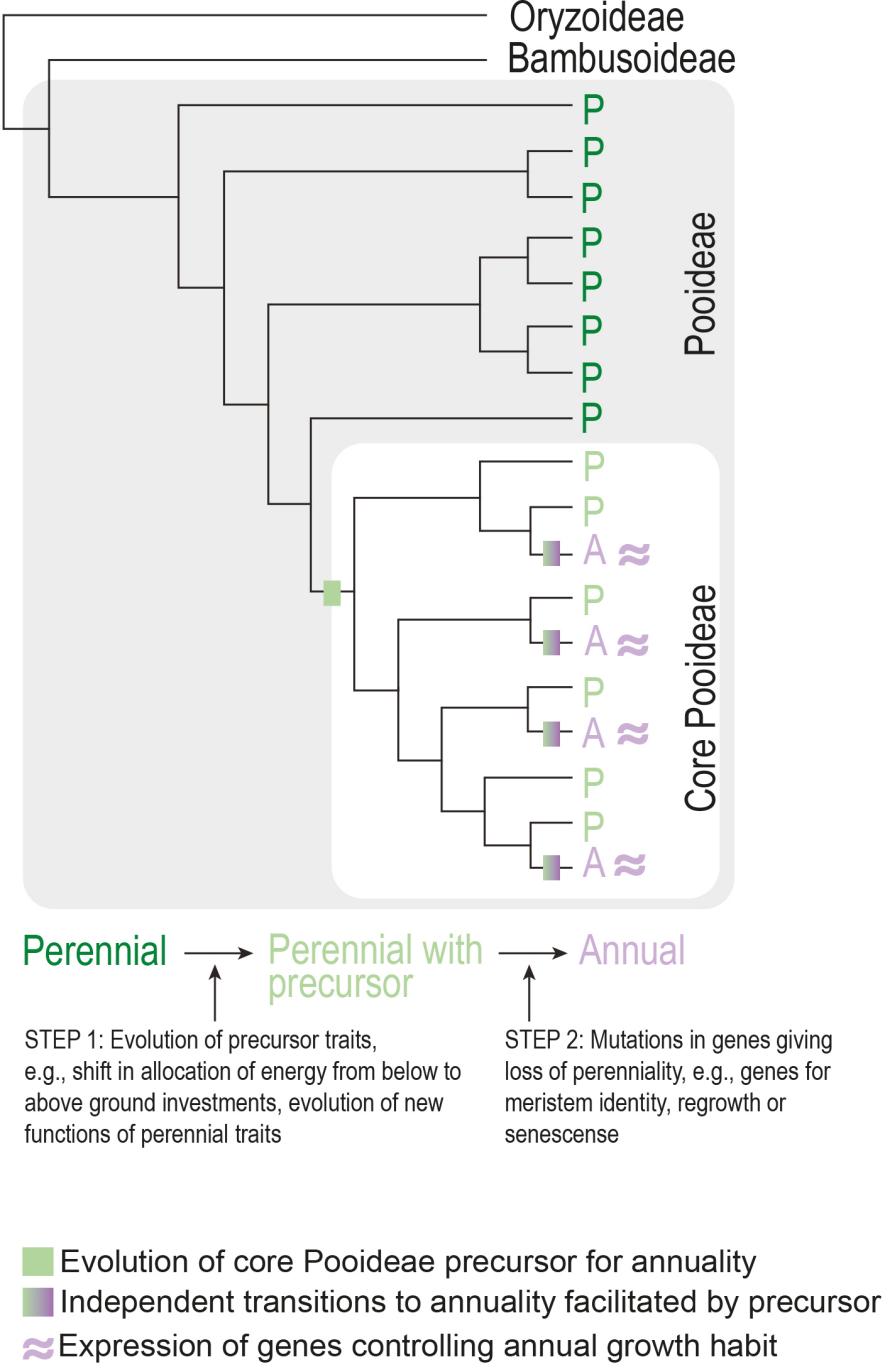
Model for the evolution of annuality in the Pooideae subfamily of grasses. Assuming the precursor represents a common genetic basis, transition to annuality occurs through parallel evolution involving the same gene(s).

Phylogenetic clustering of seemingly independently evolved traits across angiosperms is not confined to life history. A major hypothesis for the phylogenetic clustering of traits is the presence of a so-called precursor trait (see **Box 1**) in the most recent common ancestor of a clade that enables recurrent evolution of a specific phenotypic innovation (e.g., Marazzi et al., 2012; Beaulieu et al., 2013). For example, a study on the grass subfamily Pooideae found a phylogenetic pattern consistent with an evolutionary precursor in the ancestor of the core group (Lindberg et al., 2020). This could explain the unusually high number of origins of annuality in this clade. Importantly, the reconstructed pattern cannot be explained purely by which clades have or have not been exposed to the environmental selection pressures expected to lead to the evolution of annuals. In other words, several perennials outside the core clade have encountered the same warm, arid environments as the core clade, but without evolving annuality (Lindberg et al., 2020). Although it remains to be seen what might have constituted the evolutionary precursor trait(s), a strong candidate is a shift in allocation of resources from below to aboveground structures (Lindberg et al., 2020), which is an established trait linked to annuality (Grime and Hunt, 1975). Other traits, such as changes in the timing of developmental transitions, are also good precursor candidates. The precursor model predicts a similar genetic basis of annual growth habit between independently derived core Pooideae species (**Figure 6**) that warrants testing in the future.

In terms of what constitutes the genetic basis for a precursor, one or a few genes with pleiotropic effects where simple mutations affect several traits constituting annual or perennial life history strategies, would be candidates. Friedman et al. (2015) worked with perennial and annual populations of *Erythranthe guttata* (syn. *Mimulus guttatus*) and found a strong correlation between both developmental (e.g., flowering time) and growth traits (e.g., stolon production and length) with pleiotropic QTLs underlying these differences. Such QTLs are candidates for precursors, as simple mutations have large effects on the suit of traits that define life history strategy. Whether such QTLs or genes exist in larger phylogenetic groups and contribute to repeated evolution of annual life history strategies remains to be tested.

We are not aware of any other studies of evolutionary precursors for evolution of life history traits. However, attempts have been made to compare independently evolved annual/perennial species pairs to identify common genetic variation in annual versus perennial species that would be indicative of a common genetic foundation. For example, a comparison of sequence variation in *FLC* from independently evolved *Arabidopsis/Arabis* annual species identified regions in the first intron that are lower in genetic variation than expected by chance (Kiefer et al., 2017). Variation in two regulatory regions also correlates with *FLC* expression patterns in annual versus perennial species, thus indicating a common, genetic foundation of life history. The exact effect and function of the variation is unknown, but it implies a recurrent deactivation of a perennial trait.

More in-depth phylogenetic studies will help us understand if parallel mechanisms underlie convergent origins of life history traits within and across different plant families. Many of the traits involved in the annuality-perenniality syndrome, such as growth rates, are not exclusively related to life history, but their high lability might be an easy target on which selection can repeatedly act. On the other hand, in most investigated cases, annuals evolve from perennials, with no reversals to perenniality, indicating that loss of perenniality is not easily reversed through mutations (Kiefer et al., 2017). Groups that do show switches from annuality to perenniality will thus be interesting comparisons for future work, perhaps revealing selection on traits different from those driving the more common perennial to annual transitions.

## Conclusions and future directions

Annuality and perenniality are adaptive strategies that comprise fundamentally different physiological and developmental traits. Annuals have less developed root systems than perennials, making them less efficient than perennials at water and nutrient uptake; relatively less resistant to weeds and pests; and requiring yearly sowing, leading to a higher tillage requirement. One strategy for increasing sustainability in our agricultural systems is to breed perennial traits into our largely annual crop species (Crews et al., 2018). However, progress in breeding perenniality into annuals has been hampered by limited knowledge of the developmental, physiological and genetic mechanisms underlying growth habits in different plant groups, as well as potential trade-offs between production and longevity. A notable exception is the ‘perennial rice’ hybrid that has consistent yield across five years of growth in southern China (Zhang et al., 2022). The fact that annuals and perennials are often closely related suggests that only small genetic differences differentiate them (van Kleunen, 2007). Furthermore, parity can be influenced by growth environment and mechanical cropping (e.g. ratooning). Together, these findings are encouraging for the search for underlying mechanisms for life history variation and lifetime yield, since comparative studies between close relatives are less affected by the confounding signals of long, independent evolutionary trajectories.

There is no doubt that annuality has evolved convergently in different lineages of flowering plants. Nevertheless, very few comparative studies that account for phylogeny exist, making it difficult to identify and compare the mechanisms underlying convergent transitions in different groups. Although critical knowledge has been gleaned from a handful of model annual species on the control of phenological, growth, architectural, and senescence traits, we suggest that much of the variation involved in perennial to annual transitions lies upstream of these core pathways. To reveal these differences, we suggest expanding studies from annual models to their perennial sister species; for instance through comparative studies of candidate traits and genes, or by generating and studying hybrids between closely related annual and perennial species (e.g., perennial rice). Furthermore, we call for more comparative analyses in non-model angiosperms. With more detailed species sampling, the phylogenetic distribution of perennial to annual transitions, and potential reversions, as well as any environmental and developmental/genetic/physiological correlates, can be determined within certain clades. This may well indicate that the convergent evolution of annuality has largely been facilitated by evolutionary precursors, as has been demonstrated for the cool-season grasses in subfamily Pooideae. A deeper understanding of the internal and external factors that drive the evolution of annual and perennial traits will ultimately be crucial for development of sustainable food crop production in the future.

## Author contributions

All authors contributed to the writing of the manuscript. AMH and KK compiled and analyzed the data for the phylogenetic distribution of annual species in **Figure 1**. All authors contributed to the article and approved the submitted version.

## Footnotes

1 Exact numbers and phylogenetic distribution depend how exactly 'annuals' have been defined. There are 'obligate annuals', which constitute the majority of the species recognized in the literature. These can be both terrestrial and aquatic. 'Facultative annuals' can behave as either annuals or perennials (varying over space and/or time) and constitute about 20% of species categorised as annuals in the literature. Finally, there are 'biennials', which are semelparous plants that live for two growing seasons, or a single growing season but germinate in autumn instead of spring (which makes them equivalent to 'winter annuals'). These constitute about 6% of the species that can be considered annuals and are particularly prevalent in Brassicaceae and Scrophulariaceae.

## References

[B1] Abbasi J. Xu J. Dehghani H. Luo M. C. Deal K. R. McGuire P. E.. (2020). Introgression of perennial growth habit from *Lophopyrum elongatum* into wheat. Theor. Appl. Genet. 133, 2545–2554. doi: 10.1007/s00122-020-03616-x 32494869

[B2] Ahsan M. U. Hayward A. Irihimovitch V. Fletcher S. Tanurdzic M. Pocock A.. (2019). Juvenility and vegetative phase transition in tropical/subtropical tree crops. Front. Plant Sci. 10, 729. doi: 10.3389/fpls.2019.00729 31214234 PMC6558100

[B3] Albani M. C. Castaings L. Wötzel S. Mateos J. L. Wunder J. Wang R.. (2012). PEP1 of *Arabis alpina* is encoded by two overlapping genes that contribute to natural genetic variation in perennial flowering. PloS Genet. 8, e1003130. doi: 10.1371/journal.pgen.1003130 23284298 PMC3527215

[B4] Alonso-Blanco C. Aarts M. G. M. Bentsink L. Keurentjes J. J. B. Reymond M. Vreugdenhil D.. (2009). What has natural variation taught us about plant development, physiology and adaptation? Plant Cell 21, 1877–1896. doi: 10.1105/tpc.109.068114 19574434 PMC2729614

[B5] Ames O. (1939). Economic annuals and human cultures (Cambridge, Massachusetts, USA: Botanical Museum of Harvard University).

[B6] Atkinson R. R. L. Mockford E. J. Bennett C. Christin P.-A. Spriggs E. L. Freckleton R. P.. (2016). C4 photosynthesis boosts growth by altering physiology, allocation and size. Nat. Plants 2, 16038. doi: 10.1038/nplants.2016.38 27243645

[B7] Aukerman M. J. Sakai H. (2003). Regulation of flowering time and floral organ identity by a MicroRNA and its *APETALA2-*like target genes. Plant Cell 15, 2730–2741. doi: 10.1105/tpc.016238 14555699 PMC280575

[B8] Axtell M. J. Bowman J. L. (2008). Evolution of plant microRNAs and their targets. Trends Plant Sci. 13, 343–349. doi: 10.1016/j.tplants.2008.03.009 18502167

[B9] Balanzá V. Martínez-Fernández I. Sato S. Yanofsky M. Kaufmann K. Angenent G. C.. (2018). Genetic control of meristem arrest and life span in *Arabidopsis* by a *FRUITFULL-APETALA2* pathway. Nat. Commun. 9, 565. doi: 10.1038/s41467-018-03067-5 29422669 PMC5805735

[B10] Bazzaz F. A. Chiariello N. R. Coley P. D. Pitelka L. F. (1987). Allocating resources to reproduction and defense. new assessments of the costs and benefits of allocation patterns in plants are relating ecological roles to resource use. BioScience 37, 58–67. doi: 10.2307/1310178

[B11] Beaulieu J. M. O'Meara B. C. Donoghue M. J. (2013). Identifying hidden rate changes in the evolution of a binary morphological character: the evolution of plant habit in campanulid angiosperms. Syst. Biol. 62, 725–737. doi: 10.1093/sysbio/syt034 23676760

[B12] Bechtold N. Ellis J. Pelletier G. (1993). In planta *Agrobacterium* mediated gene transfer by infiltration of adult *Arabidopsis thaliana* plants. C. R. Acad. Sci. Paris 316, 1194–1199.

[B13] Bena G. Lejeune B. Prosperi J.-M. Olivieri I. (1998). Molecular phylogenetic approach for studying life-history evolution: the ambiguous example of the genus *Medicago* l. Proc. R. Soc Lond. B. 265, 1141–1151. doi: 10.1098/rspb.1998.0410 PMC1689169 9684377

[B14] Bergonzi S. Albani M. C. (2011). Reproductive competence from an annual and a perennial perspective. J. Exp. Bot. 62, 4415–4422. doi: 10.1093/jxb/err192 21725031

[B15] Bergonzi S. Albani M. C. Ver Loren van Themaat E. Coupland G. (2013). Mechanisms of age-dependent response to winter temperature in perennial flowering of arabis alpina. Science 340, 1094–1097. doi: 10.1126/science.1234116 23723236

[B16] Blount Z. D. Lenski R. E. Losos J. B. (2018). Contingency and determinism in evolution: replaying life’s tape. Science 362, eaam5979. doi: 10.1126/science.aam5979 30409860

[B17] Cavender-Bares J. Kozak K. H. Fine P. V. Kembel S. W. (2009). The merging of community ecology and phylogenetic biology. Ecol. Lett. 12, 693–715. doi: 10.1111/j.1461-0248.2009.01314.x 19473217

[B18] Charnov E. L. Schaffer W. M. (1973). Life-history consequences of natural selection: Cole's result revisited. Am. Nat. 107, 791–793. doi: 10.1086/282877

[B19] Cheng Y.-J. Shang G.-D. Xu Z.-G. Yu S. Wu L.-Y. Zhai D.. (2021). Cell division in the shoot apical meristem is a trigger for miR156 decline and vegetative phase transition in arabidopsis. P Natl. Acad. Sci. U.S.A. 118, e2115667118. doi: 10.1073/pnas.2115667118 PMC8609562 34750273

[B20] Choi K. Kim J. Müller S. Y. Oh M. Underwood C. Henderson I.. (2016). Regulation of MicroRNA-mediated developmental changes by the SWR1 chromatin remodelling complex. Plant Phys. 171, 1128–1143. doi: 10.1104/pp.16.00332 PMC4902616 27208270

[B21] Chuck G. Cigan A. M. Saeteurn K. Hake S. (2007). The heterochronic maize mutant Corngrass1 results from overexpression of a tandem microRNA. Nat. Genet. 39, 544–549. doi: 10.1038/ng2001 17369828

[B22] Cockcroft C. E. den Boer B. G. W. Healy J. M. S. Murray J. A. H. (2000). Cyclin d control of growth rate in plants. Nature 405, 575–579. doi: 10.1038/35014621 10850717

[B23] Corbin J. D. D'Antonio C. M. (2004). Competition between native perennial and exotic annual grasses: implications for an historical invasion. Ecology 85, 1273–1283. doi: 10.1890/02-0744

[B24] Crevillén P. Yang H. Cui X. Greeff C. Trick M. Qiu Q.. (2014). Epigenetic reprogramming that prevents transgenerational inheritance of the vernalized state. Nature 515, 587–590. doi: 10.1038/nature13722 25219852 PMC4247276

[B25] Crews T. E. DeHaan L. R. (2015). The strong perennial vision: a response. Agroecol. Sustain. Food Syst. 39, 500–515. doi: 10.1080/21683565.2015.1008777

[B26] Crews T. E. Carton W. Olsson L. (2018). Is the future of agriculture perennial? Imperatives and opportunities to reinvent agriculture by shifting from annual monocultures to perennial polycultures. Global Sustainability. 1, e11. doi: 10.1017/sus.2018.11

[B27] Cruz-Mazo G. Buide M. L. Samuel R. Narbona E. (2009). Molecular phylogeny of scorzoneroides (Asteraceae): Evolution of heterocarpy and annual habit in unpredictable environments. Mol. Phylogenet. Evol. 53, 835–847. doi: 10.1016/j.ympev.2009.08.001 19666128

[B28] Cui Y. Cheng J. Ruan S. Qi P. Liu W. Bian W.. (2020). The heterochronic gene oryza sativa LIKE HETEROCHROMATIN PROTEIN 1 modulates *miR156b/c/i/e* levels. J. Integr. Plant Biol. 62, 1839–1852. doi: 10.1111/jipb.12991 32644250

[B29] Czaban A. Sharma S. Byrne S. L. Spannagl M. Mayer K. F. Asp T. (2015). Comparative transcriptome analysis within the *Lolium/Festuca* species complex reveals high sequence conservation. BMC Genomics 16, 249. doi: 10.1186/s12864-015-1447-y 25886302 PMC4389671

[B30] Debernardi J. M. Woods D. P. Li K. Li C. Dubcovsky J. (2022). *MiR172-APETALA2*-like genes integrate vernalization and plant age to control flowering time in wheat. PloS Genet. 18, e1010157. doi: 10.1371/journal.pgen.1010157 35468125 PMC9037917

[B31] De Souza J. G. Da Silva J. V. (1987). Partitioning of carbohydrates in annual and perennial cotton (*Gossypium hirsutum* l.). J. Exp. Bot. 38, 1211–1218. doi: 10.1093/jxb/38.7.1211

[B32] Doebley J. Stec A. Gustus C. (1995). *teosinte branched1* and the origin of maize: evidence for epistasis and the evolution of dominance. Genetics 141, 333–346. doi: 10.1093/genetics/141.1.333 8536981 PMC1206731

[B33] Dong Z. Xiao Y. Govindarajulu R. Feil R. Siddoway M. L. Nielsen T.. (2019). The regulatory landscape of a core maize domestication module controlling bud dormancy and growth repression. Nat. Commun. 10, 3810. doi: 10.1038/s41467-019-11774-w 31444327 PMC6707278

[B34] Drummond C. S. (2008). Diversification of *Lupinus* (Leguminosae) in the western new world: Derived evolution of perennial life history and colonization of montane habitats. Mol. Phyl. Evol. 48, 408–421. doi: 10.1016/j.ympev.2008.03.009 18534869

[B35] Du Z. Jia X. L. Wang Y. Wu T. Han Z. H. Zhang X. Z. (2015). Redox homeostasis and reactive oxygen species scavengers shift during ontogenetic phase changes in apple. Plant Sci. 236, 283–294. doi: 10.1016/j.plantsci.2015.04.008 26025541

[B36] Estiarte M. Peñuelas J. (2015). Alteration of the phenology of leaf senescence and fall in winter deciduous species by climate change: effects of nutrient proficiency. Global Change Biol. 21, 1005–1017. doi: 10.1111/gcb.12804 25384459

[B37] Evans M. E. K. Hearn D. J. Hahn W. J. Spangle J. M. Venable D. L. (2005). Climate and life-history evolution in evening primroses (*Oenothera*, onagraceae): A phylogenetic comparative analysis. Evolution 59, 1914–1927. doi: 10.1111/j.0014-3820.2005.tb01061.x 16261729

[B38] Evans M. M. S. Poethig R. S. (1995). Gibberellins promote vegetative phase-change and reproductive maturity in maize. Plant Physiol. 108, 475–487. doi: 10.1104/pp.108.2.475 7610158 PMC157366

[B39] Exner V. Aichinger E. Shu H. Wildhaber T. Alfarano P. Caflisch A.. (2009). The chromodomain of LIKE HETEROCHROMATIN PROTEIN 1 is essential for H3K27me3 binding and function in *Arabidopsis* development. PloS One 4, e5335. doi: 10.1371/journal.pone.0005335 19399177 PMC2670505

[B40] Feng S. Xu Y. Guo C. Zheng J. Zhou B. Zhang Y.. (2016). Modulation of miR156 to identify traits associated with vegetative phase change in tobacco (*Nicotiana tabacum*). J. Exp. Bot. 67, 1493–1504. doi: 10.1093/jxb/erv551 26763975

[B41] Fjellheim S. Young D. A. Paliocha M. Johnsen S. S. Schubert M. Preston J. C. (2022). Major niche transitions in Pooideae correlate with variation in photoperiodic flowering and evolution of CCT domain genes. J. Exp. Bot. 73, 4079–4093. doi: 10.1093/jxb/erac149 35394528 PMC9232202

[B42] Fouracre J. He J. Chen V. Sidoli S. Poethig R. (2021). VAL genes regulate vegetative phase change *via* miR156-dependent and independent mechanisms. PloS Genet. 17, e1009626. doi: 10.1371/journal.pgen.1009626 34181637 PMC8270478

[B43] Franco M. Silvertown J. (1996). Life history variation in plants: an exploration of the fast-slow continuum hypothesis. Philos. T. R. Soc Lon. B. 351, 1341–1348. doi: 10.1098/rstb.1996.0117

[B44] Friedman J. (2020). The evolution of annual and perennial plant life histories: ecological correlates and genetic mechanisms. Annu. Rev. Ecol. Evol. Syst. 51, 461–481. doi: 10.1146/annurev-ecolsys-110218-024638

[B45] Friedman J. Twyford A. D. Willis J. H. Blackman B. K. (2015). The extent and genetic basis of phenotypic divergence in life history traits in *Mimulus guttatus* . Mol. Ecol. 24, 111–122. doi: 10.1111/mec.13004 25403267 PMC4657477

[B46] García-Camacho R. Metz J. Bilton M. C. Tielbörger K. (2017). Phylogenetic structure of annual plant communities along an aridity gradient. interacting effects of habitat filtering and shifting plant–plant interactions. Isr. J. Plant Sci. 64, 122–134. doi: 10.1080/07929978.2017.1288477

[B47] Garnier E. (1992). Growth analysis of congeneric annual and perennial grass species. J. Ecol. 80, 665–675. doi: 10.2307/2260858

[B48] Garnier E. Cordonnier P. Guillerm J. L. Sonie L. (1997). Specific leaf area and leaf nitrogen concentration in annual and perennial grass species growing in Mediterranean old-fields. Oecologia 111, 490–498. doi: 10.1007/s004420050262 28308109

[B49] Garnier E. Laurent G. (1994). Leaf anatomy, specific mass and water-content in congeneric annual and perennial grass species. New Phytol. 128, 725–736. doi: 10.1111/j.1469-8137.1994.tb04036.x

[B50] Garnier E. Vancaeyzeele S. (1994). Carbon and nitrogen content of congeneric annual and perennial grass species – relationships with growth. Plant Cell Environ. 17, 399–407. doi: 10.1111/j.1365-3040.1994.tb00308.x

[B51] Gaudin V. Libault. M. Pouteau S. Juul T. Zhao G. Lefebvre D.. (2001). Mutations in *LIKE HETEROCHROMATIN PROTEIN 1* affect flowering time and plant architecture in arabidopsis. Development 128, 4847–4858. doi: 10.1242/dev.128.23.4847 11731464

[B52] Gould S. J. (1989). Wonderful life: The burgess shale and the nature of history (New York, USA: Norton and Company).

[B53] Grime J. P. Hunt R. (1975). Relative growth-rate: its range and adaptive significance in a local flora. J. Ecol. 63, 393–422. doi: 10.2307/2258728

[B54] Guo C. Jiang Y. Shi M. Wu X. Wu G. (2021). *ABI5* acts downstream of *miR159* to delay vegetative phase change in arabidopsis. New Phyt. 231, 339–350. doi: 10.1111/nph.17371 33774835

[B55] Guo C. Xu Y. Shi M. Lai Y. Wu X. Wang H.. (2017). Repression of miR156 by *miR159* regulates the timing of the juvenile-to-adult transition in *Arabidopsis* . Plant Cell 29, 1293–1304. doi: 10.1105/tpc.16.00975 28536099 PMC5502449

[B56] Han J. Zhang T. Li J. Hu Y. (2021). Identification of miRNA responsive to early flowering in tree peony (*Paeonia osti*i) by high-throughput sequencing. J. Hortic. Sci. Biotech. 96, 297–310. doi: 10.1080/14620316.2020.1846466

[B57] Hashimoto S. Tezuka T. Yokoi S. (2019). Morphological changes during juvenile-to-adult phase transition in sorghum. Planta 250, 1557–1566. doi: 10.1007/s00425-019-03251-x 31359138

[B58] Heidel A. J. Kiefer C. Coupland G. Rose L. E. (2016). Pinpointing genes underlying annual/perennial transitions with comparative genomics. BMC Genomics 17, 921. doi: 10.1186/s12864-016-3274-1 27846808 PMC5111240

[B59] Herron S. A. Rubin M. J. Ciotir C. Crews T. E. Van Tassel D. L. Miller A. J. (2020). Comparative analysis of early life stage traits in annual and perennial *Phaseolus* crops and their wild relatives. Front. Plant Sci. 11, 34. doi: 10.3389/fpls.2020.00034 32210978 PMC7076113

[B60] Heyduk K. Moreno-Villena J. J. Gilman I. S. Christin P.-A. Edwards E. J. (2019). The genetics of convergent evolution: insights from plant photosynthesis. Nat. Rev. Genet. 20, 485–493. doi: 10.1038/s41576-019-0107-5 30886351

[B61] Hileman L. C. (2014). Trends in flower symmetry evolution revealed through phylogenetic and developmental genetic advances. Phil. Trans. R. Soc B. 369, 20130348. doi: 10.1098/rstb.2013.0348 24958922 PMC4071522

[B62] Hoogendoorn J. (1984). A comparison of different vernalization techniques in wheat (*Triticum aestivum* l.). J. Plant Physiol. 116, 11–20. doi: 10.1016/S0176-1617(84)80079-6 23194873

[B63] Hughes P. W. Soppe W. J. J. Albani M. C. (2019). Seed traits are pleiotropically regulated by the flowering time gene *PERPETUAL FLOWERING 1 (PEP1)* in the perennial *Arabis alpina* . Mol. Ecol. 28, 1183–1201. doi: 10.1111/mec.15034 30712274 PMC6850658

[B64] Humphreys A. M. Antonelli A. Pirie M. D. Linder H. P. (2011). Ecology and evolution of the diaspore “burial” syndrome. Evolution 65, 1163–1180. doi: 10.1111/j.1558-5646.2010.01184.x 21062276

[B65] Hu F. Y. Tao D. Y. Sacks E. Fu B. Y. Xu P. Li J.. (2003). Convergent evolution of perenniality in rice and sorghum. Proc. Natl. Acad. Sci. U.S.A. 100, 4050–4054. doi: 10.1073/pnas.0630531100 12642667 PMC153046

[B66] Hu H. Mauro-Herrera M. Doust A. N. (2018). Domestication and Improvement in the Model C4 Grass, Setaria. Frontiers in Plant Science, 9. doi: 10.3389/fpls.2018.00719 PMC5986938 29896214

[B67] Hyun Y. Richter R. Coupland G. (2017). Competence to flower: age-controlled sensitivity to environmental cues. Plant Phys. 173, 36–46. doi: 10.1104/pp.16.01523 PMC5210750 27920161

[B68] Hyun Y. Vincent C. Tilmes V. Bergonzi S. Kiefer C. Coupland G. (2019). A regulatory circuit conferring varied flowering response to cold in annual and perennial plants. Science 363 (6425), 409–412. doi: 10.1126/science.aau8197 30679374

[B69] Igea J. Miller E. F. Papadopulos A. S. T. Tanentzap A. J. (2017). Seed size and its rate of evolution correlate with species diversification across angiosperms. PloS Biol. 15, e2002792. doi: 10.1371/journal.pbio.2002792 28723902 PMC5536390

[B70] Jeffrey E. C. (1916). The anatomy of woody plants (Chicago, Illinois, USA: University Press).

[B71] Jones T. A. Zhang X. Y. Wang R. R. C. (1999). Genome characterization of MT-2 perennial and OK-906 annual wheat x intermediate wheatgrass hybrids. Crop Sci. 39, 1041–1043. doi: 10.2135/cropsci1999.0011183X003900040013x

[B72] Kadereit G. Mucina L. Freitag H. (2006). Phylogeny of *Salicornioideae* (Chenopodiaceae): diversification, biogeography, and evolutionary trends in leaf and flower morphology. Taxon 55, 617–642. doi: 10.2307/25065639

[B73] Keeley J. E. Bond W. J. (1999). Mast flowering and semelparity in bamboos: the bamboo fire cycle hypothesis. Am. Nat. 154, 383–391. doi: 10.1086/303243 10506551

[B74] Kiefer C. Severing E. Karl R. Bergonzi S. Koch M. Tresch A.. (2017). Divergence of annual and perennial species in the brassicaceae and the contribution of cis-acting variation at *FLC* orthologues. Mol. Ecol. 26, 3437–3457. doi: 10.1111/mec.14084 28261921 PMC5485006

[B75] Körner C. Larcher W. (1988). “Plant life in cold climates,” in Plants and temperature. Eds. Long S. Woodward F. (Cambridge: The Company of Biol Ltd), 25–57. 3270208

[B76] Kumari R. Chaudhary S. Mishra R. K. Rai S. P. Rai S. K. Sharma V.. (2010). Regulation of lifespan by the *LLI* and *EGD* genes in the perennial plant species *Catharanthus roseus* . P. Indian Acad. Sci. 76, 27–39.

[B77] Kumar K. Kondhare R. K. Vetal P. V. Banerjee A. K. (2020). PcG proteins MSI1 and BMI1 function upstream of *miR156* to regulate aerial tuber formation in potato. Plant Phys. 182, 185–203. doi: 10.1104/pp.19.00416 PMC6945842 31427464

[B78] Lammer D. Cai X. Arterburn M. Chatelain J. Murray T. Jones S. (2004). A single chromosome addition from *Thinopyrum elongatum* confers a polycarpic, perennial habit to annual wheat. J. Exp. Bot. 55, 1715–1720. doi: 10.1093/jxb/erh209 15234999

[B79] Lazaro A. Obeng-Hinneh E. Albani M. C. (2018). Extended vernalization regulates inflorescence fate in *Arabis alpina* by stably silencing *PERPETUAL FLOWERING1* . Plant Phys. 176, 2819–2833. doi: 10.1104/pp.17.01754 PMC5884582 29467177

[B80] Lim P. O. Kim H. J. Nam H. G. (2007). Leaf senescence. Annu. Rev. Plant Biol. 58, 115–136. doi: 10.1146/annurev.arplant.57.032905.105316 17177638

[B81] Lindberg C. L. Hanslin H. M. Schubert M. Marcussen T. Trevaskis B. Preston J. C.. (2020). Increased above-ground resource allocation is a likely precursor for independent evolutionary origins of annuality in the pooideae grass subfamily. New Phytol. 228, 318–329. doi: 10.1111/nph.16666 32421861

[B82] Liu Q. Su Y. Zhu Y. Peng K. Hong B. Wang R.. (2019). Manipulating osa-MIR156f expression by D18 promoter to regulate plant architecture and yield traits both in seasonal and ratooning rice. Biol. Proc. Online 21, 21. doi: 10.1186/s12575-019-0110-4 PMC6827258 31700499

[B83] Li H. Zhao X. Dai H. Wu W. Mao W. Zhang Z. (2012). Tissue culture responsive microRNAs in strawberry. Plant Mol. Biol. Rep. 30, 1047–1054. doi: 10.1007/s11105-011-0406-2

[B84] Losos J. B. (2011). Convergence, adaptation and constraint. Evolution 65, 1827–1840. doi: 10.1111/j.1558-5646.2011.01289.x 21729041

[B85] Magallón S. Gomez-Acevedo S. Sanchez-Reyes L. L. Hernandez-Hernandez T. (2015). A metacalibrated time-tree documents the early rise of flowering plant phylogenetic diversity. New Phytol. 207, 437–453. doi: 10.1111/nph.13264 25615647

[B86] Mahler D. L. Ingram T. Revell L. J. Losos J. B. (2013). Exceptional convergence on the macroevolutionary landscape in island lizard radiations. Science 341, 292–295. doi: 10.1126/science.1232392 23869019

[B87] Malik N. S. A. Berrie A. M. M. (1975). Correlative effects of fruits and leaves in senescence of pea plants. Planta 124, 169–175. doi: 10.1007/BF00384759 24435234

[B88] Ma A. Qiu Y. Raihan T. Paudel B. Dahal S. Zhuang Y.. (2019). The genetics and genome-wide screening of regrowth loci, a key component of perennialism in *Zea diploperennis.* G3 genes. Genom. Genet. 9, 1393–1403. doi: 10.1534/g3.118.200977 PMC6505134 30808689

[B89] Marazzi B. Ané C. Simon M. F. Delgado-Salinas A. Luckow M. Sanderson M. J. (2012). Locating evolutionary precursors on a phylogenetic tree. Evolution 66, 3918–3930. doi: 10.1111/j.1558-5646.2012.01720.x 23206146

[B90] Massante J. C. Köbel M. Pinho P. Gerhold P. Branquinho C. Nunes A. (2021). Phylogenetic structure of understorey annual and perennial plant species reveals opposing responses to aridity in a Mediterranean biodiversity hotspot. Sci. Total Environ. 761, 144018. doi: 10.1016/j.scitotenv.2020.144018 33352349

[B91] Matts J. Jagadeeswaran G. Roe B. A. Sunkar R. (2010). Identification of microRNAs and their targets in switchgrass, a model biofuel plant species. J. Plant Physiol. 167, 896–904. doi: 10.1016/j.jplph.2010.02.001 20207044

[B92] Ma J. Zhao P. Liu S. Yang Q. Guo H. (2020). The control of developmental phase transitions by microRNAs and their targets in seed plants. Int. J. Mol. Sci. 21, 1971. doi: 10.3390/ijms21061971 32183075 PMC7139601

[B93] Michaels S. D. Amasino R. M. (1999). *FLOWERING LOCUS c* encodes a novel MADS domain protein that acts as a repressor of flowering. Plant Cell 11, 949. doi: 10.1105/tpc.11.5.949 10330478 PMC144226

[B94] Minina E. A. Sanchez-Vera V. Moschou P. N. Suarez M. F. Sundberg E. Weih M.. (2013). Autophagy mediates caloric restriction-induced lifespan extension in *Arabidopsis* . Aging Cell 12, 327–329. doi: 10.1111/acel.12048 23331488

[B95] Miryeganeh M. (2021). Senescence: the compromised time of death that plants may call on themselves. Genes 12, 143. doi: 10.3390/genes12020143 33499161 PMC7912376

[B96] Monroe J. G. Gill B. Turner K. G. McKay J. K. (2019). Drought regimens predict life history strategies in *Heliophila* . New Phytol. 223, 2054–2062. doi: 10.1111/nph.15919 31087648

[B97] Montiglio P.-O. Dammhahn M. Messier G. D. Reale D. (2018). Pace-of-life syndrome revisited: the role of ecological conditions and natural history on the slow-fast continuum. Behav. Ecol. Sociobiol. 72, 116. doi: 10.1007/s00265-018-2526-2

[B98] Munne-Bosch S. (2008). Do perennials really senesce? Trends Plant Sci. 13, 216–220. doi: 10.1016/j.tplants.2008.02.002 18328774

[B99] Nishio H. Nagano A. J. Ito T. Suzuki Y. Kudoh H. (2020). Seasonal plasticity and diel stability of H3K27me3 in natural fluctuating environments. Nat. Plants 6, 1091–1097. doi: 10.1038/s41477-020-00757-1 32868888

[B100] Nooden L. D. Leopold A. C. (1988). Senescence and aging in plants (San Diego, CA, USA: Academic Press).

[B101] Nunes A. Köbel M. Pinho P. Matos P. Costantini E. A. C. Soares C.. (2019). Local topographic and edaphic factors largely predict shrub encroachment in Mediterranean drylands. Sci. Total Environ. 657, 310–318. doi: 10.1016/j.scitotenv.2018.11.475 30543980

[B102] Ogburn M. R. Edwards E. J. (2015). Life history lability underlies rapid climate niche evolution in the angiosperm clade montiaceae. Mol. Phylogenet. Evol. 92, 181–192. doi: 10.1016/j.ympev.2015.06.006 26143714

[B103] Park J.-Y. Kim H. Lee I. (2017). Comparative analysis of molecular and physiological traits between perennial *Arabis alpina* pajares and annual *Arabidopsis thaliana* sy-0. Sci. Rep. 7, 13348. doi: 10.1038/s41598-017-13606-7 29042663 PMC5645391

[B104] Pettit N. E. Froend R. H. Ladd P. G. (1995). Grazing in remnant woodland vegetation: changes in species composition and life form groups. J. Veg. Sci. 6, 121–130. doi: 10.2307/3236263

[B105] Pico S. Ortiz-Marchena M. I. Merini W. Calonje M. (2015). Deciphering the role of *Polycomb repressive complex 1 (PRC1)* variants in regulating the acquisition of flowering competence in *Arabidopsis* . Plant Phys. 168, 1286–1297. doi: 10.1104/pp.15.00073 PMC4528732 25897002

[B106] Plucknett D. L. Evenson J. P. Sanford W. G. (1970). Ratoon cropping. Adv. Agron. 22, 285–330. doi: 10.1016/S0065-2113(08)60271-0

[B107] Poethig R. S. (2003). Phase change and the regulation of developmental timing in plants. Science 301, 334–336. doi: 10.1126/science.1085328 12869752

[B108] Ponraj U. Theres K. (2020). Keep a distance to be different: axillary buds initiating at a distance from the shoot apical meristem are crucial for the perennial lifestyle of *Arabis alpina* . New Phytol. 227, 116–131. doi: 10.1111/nph.16512 32112411

[B109] Poorter H. Niinemets Ü. Poorter L. Wright I. J. Villar R. (2009). Causes and consequences of variation in leaf mass per area (LMA): a meta-analysis. New Phytologist 182(3), 565–588. doi: 10.1111/j.1469-8137.2009.02830.x 19434804

[B110] Preston J. C. Fjellheim S. (2020). Understanding past, and predicting future, niche transitions based on grass flowering time variation. Plant Phys. 183, 822–839. doi: 10.1104/pp.20.00100 PMC7333695 32404414

[B111] Preston J. C. Fjellheim S. (2022). Flowering time runs hot and cold. Plant Phys. 190, 5–18. doi: 10.1093/plphys/kiac111 PMC9434294 35274728

[B112] Preston J. C. Sandve S. R. (2013). Adaptation to seasonality and the winter freeze. Front. Plant Sci. 4, 167. doi: 10.3389/fpls.2013.00167 23761798 PMC3669742

[B113] Qiu K. Li Z. Chen J. Wu S. Zhu X. Gao S.. (2015). *EIN3* and *ORE1* accelerate degreening during ethylene-mediated leaf senescence by directly activating chlorophyll catabolic genes in *Arabidopsis* . PloS Genet. 11, e1005399. doi: 10.1371/journal.pgen.1005399 26218222 PMC4517869

[B114] Roumet C. Urcelay C. Díaz S. (2006). Suites of root traits differ between annual and perennial species growing in the field. New Phytol. 170, 357–368. doi: 10.1111/j.1469-8137.2006.01667.x 16608460

[B115] Salguero-Gomez R. Jones O. R. Jongejans E. Blomberg S. P. Hodgson D. J. Mbeau-Ache C.. (2016). Fast-slow continuum and reproductive strategies structure plant life-history variation worldwide. Proc. Natl. Acad. Sci. U.S.A. 113, 230–235. doi: 10.1073/pnas.1506215112 26699477 PMC4711876

[B116] Sasani S. Hemming M. N. Oliver S. N. Greenup A. Tavakkol-Afshari R. Mahfoozi S.. (2009). The influence of vernalization and daylength on expression of flowering-time genes in the shoot apex and leaves of barley (*Hordeum vulgare*). J. Exp. Bot. 60, 2169–2178. doi: 10.1093/jxb/erp098 19357429 PMC2682508

[B117] Schwartz C. J. Doyle M. R. Manzaneda A. J. Rey P. J. Mitchell-Olds T. Amasino R. M. (2010). Natural variation of flowering time and vernalization responsiveness in *Brachypodium distachyon* . Bioenergy Res. 3, 38–46. doi: 10.1007/s12155-009-9069-3

[B118] Serivichyaswat P. Ryu H. S. Kim W. Kim S. Chung K. S. Kim J. J.. (2015). Expression of the floral repressor miRNA156 is positively regulated by the AGAMOUS-like proteins AGL15 and AGL18. Mol. Cells 38, 259–266. doi: 10.14348/molcells.2015.2311 25666346 PMC4363726

[B119] Sgamma T. Jackson A. Muleo R. Thomas B. Massiah A. (2014). *TEMPRANILLO* is a regulator of juvenility in plants. Sci. Rep. 4, 3704. doi: 10.1038/srep03704 24424565 PMC3892441

[B120] Sheldon C. C. Hills M. J. Lister C. Dean C. Dennis E. S. Peacock W. J. (2008). Resetting of *FLOWERING LOCUS c* expression after epigenetic repression by vernalization. Proc. Natl. Acad. Sci. U.S.A. 105, 2214–2219. doi: 10.1073/pnas.0711453105 18250331 PMC2542874

[B121] Shin D. Lee S. Kim T. H. Lee J. H. Park J. Lee J.. (2020). Natural variations at the *Stay-green* gene promoter control lifespan and yield in rice cultivars. Nat. Commun. 11, 2819. doi: 10.1038/s41467-020-16573-2 32499482 PMC7272468

[B122] Silva P. O. Batista D. S. Cavalcanti J. H. F. Koehler A. D. Vieira L. M. Fernandes A. M.. (2019). Leaf heteroblasty in *Passiflora edulis* as revealed by metabolic profiling and expression analyses of the microRNAs *miR156* and *miR172* . Ann. Bot. 123, 1191–1203. doi: 10.1093/aob/mcz025 30861065 PMC6612941

[B123] Soppe W. J. J. Torre N. V. Albani M. C. (2021). The diverse roles of FLOWERING LOCUS c in annual and perennial brassicaceae species. Front. Plant Sci. 12, 627258. doi: 10.3389/fpls.2021.627258 33679840 PMC7927791

[B124] Stearns S. C. (1992). The evolution of life histories (Oxford, UK: Oxford University Press).

[B125] Stebbins G. L. (1957). Self fertilisation and population variability in the higher plants. Amer. Nat. 861, 337–354. doi: 10.1086/281999

[B126] Stebbins G. L. (1982). Perspectives in evolutionary theory. Evolution 36, 1109–1118. doi: 10.1111/j.1558-5646.1982.tb05482.x 28563574

[B127] Stuart Y. E. (2019). Divergent uses of “parallel evolution” during the history of *The American naturalist* . Am. Nat. 193, 11–19. doi: 10.1086/700718 30624101

[B128] Swentowsky K. W. Bell H. S. Wills D. M. Dawe R. K. (2021). QTL map of early- and late-stage perennial regrowth in *Zea diploperennis* . Front. Plant Sci. 12, 707839. doi: 10.3389/fpls.2021.707839 34504508 PMC8421791

[B129] Tanaka N. (2012). Gibberellin is not a regulator of miR156 in rice juvenile-adult phase change. Rice 5, 25. doi: 10.1186/1939-8433-5-25 24279896 PMC4883733

[B130] Tank D. C. Olmstead R. G. (2008). From annuals to perennials: phylogeny of subtribe *Castillejinae* (Orobanchaceae). Am. J. Bot. 95, 608–625. doi: 10.3732/ajb.2007346 21632387

[B131] Telfer A. Bollman K. M. Poethig R. S. (1997). Phase change and the regulation of trichome distribution in *Arabidopsis thaliana* . Development 124, 645–654. doi: 10.1242/dev.124.3.645 9043079

[B132] Teotia S. Tang G. (2014). To bloom or not to bloom: role of MicroRNAs in plant flowering. Mol. Plant 8, 359–377. doi: 10.1016/j.molp.2014.12.018 25737467

[B133] Thomas H. M. (1995). Meiosis of triploid *Lolium.* II. discrepancies between the analyses of chromosome configurations at metaphase I in inverse autoallotriploid combinations. Heredity 75, 446–452. doi: 10.1038/hdy.1995.160

[B134] Thomas H. (2013). Senescence, ageing and death of the whole plant. New Phytol. 197, 696–711. doi: 10.1111/nph.12047 23176101

[B135] Thomas H. M. Thomas H. M. Ougham H. (2000). Annuality, perenniality and cell death. J. Exp. Bot. 51, 1781–1788. doi: 10.1093/jexbot/51.352.1781 11113157

[B136] Tofts R. J. (2004). *Geranium robertianum* l. J. Ecol. 92, 537–555. doi: 10.1111/j.0022-0477.2004.00892.x

[B137] Tripathi R. K. Bregitzer P. Singh J. (2018). Genome-wide analysis of the SPL/miR156 module and its interaction with the AP2/miR172 unit in barley. Sci. Rep. 8, 7085. doi: 10.1038/s41598-018-25349-0 29728569 PMC5935748

[B138] van Kleunen M. (2007). Adaptive genetic differentiation in life-history traits between populations of *Mimulus guttatus* with annual and perennial life-cycles. Evol. Ecol. 21, 185–199. doi: 10.1007/s10682-006-0019-7

[B139] Vasseur F. Exposito-Alonso M. Ayala-Garay O. J. Wang G. Enquist B. J. Vile D.. (2018). Adaptive diversification of growth allometry in the plant *Arabidopsis thaliana* . Proc. Natl. Acad. Sci. U.S.A. 115, 3416–3421. doi: 10.1073/pnas.1709141115 29540570 PMC5879651

[B140] Vico G. Manzoni S. Nkurunziza L. Murphy K. Weih M. (2016). Trade-offs between seed output and life span – a quantitative comparison of traits between annual and perennial congeneric species. New Phytol. 209, 104–114. doi: 10.1111/nph.13574 26214792

[B141] von Bothmer R. Flink J. Jacobsen N. Kotimaki M. Landstrom T. (1983). Interspecific hybridization with cultivated barley (*Hordeum vulgare* l.). Hereditas 99, 219–244. doi: 10.1111/j.1601-5223.1983.tb00895.x 6668209

[B142] Wang R. Farrona S. Vincent C. Joecker A. Schoof H. Turck F.. (2009). PEP1 regulates perennial flowering in *Arabis alpina* . Nature 459 (7245), 423–427. doi: 10.1038/nature07988 19369938

[B143] Wang J.-W. Park M. Y. Wang L.-J. Koo Y. Chen X.-Y. Weigel D.. (2011). MiRNA control of vegetative phase change in trees. PloS Genet. 7, e1002012. doi: 10.1371/journal.pgen.1002012 21383862 PMC3044678

[B144] Wang H. Wang H. (2015). The miR156/SPL module, a regulatory hub and versatile toolbox, gears up crops for enhanced agronomic traits. Mol. Plant 8, 677–688. doi: 10.1016/j.molp.2015.01.008 25617719

[B145] Ware A. Walker C. H. Simura J. Gonzalez-Suarez P. Ljung K. Bishopp A.. (2020). Auxin exports from proximal fruits drives arrest in temporally competent inflorescence. Nat. Plants. 6, 699–707. doi: 10.1038/s41477-020-0661-z 32451444

[B146] WCSP (2018) World checklist of selected plant families. Available at: http://wcsp.science.kew.org/.

[B147] Wei Q. Ma C. Xu Y. Wang T. Chen Y. Lü J.. (2017). Control of chrysanthemum flowering through integration with an aging pathway. Nat. Commun. 8, 829. doi: 10.1038/s41467-017-00812-0 29018260 PMC5635119

[B148] Whipple C. J. Kebrom T. H. Weber A. L. Yang F. Hall D. Meeley R.. (2011). Grassy tillers1 promotes apical dominance in maize and responds to shade signals in the grasses. Proc. Natl. Acad. Sci. U.S.A. 108, E506–E512. doi: 10.1073/pnas.1102819108 21808030 PMC3158142

[B149] Whittaker C. Dean C. (2017). The *FLC* locus: a platform for discoveries in epigenetics and adaptation. Annu. Rev. Cell Dev. Bi. 33, 555–575. doi: 10.1146/annurev-cellbio-100616-060546 28693387

[B150] Whyte R. O. (1977). The botanical neolithic revolution. Hum. Ecol. 5, 209–222. doi: 10.1007/BF00891278

[B151] Wills D. M. Whipple C. J. Takuno S. Kursel L. E. Shannon L. M. Ross-Ibarra J.. (2013). From many, one: genetic control of prolificacy during maize domestication. PloS Genet. 9, e1003604. doi: 10.1371/journal.pgen.1003604 23825971 PMC3694832

[B152] Wu X. Y. Kuai B. K. Jia J. Z. Jing H. C. (2012). Regulation of leaf senescence and crop genetic improvement. J. Integr. Plant Biol. 54, 936–952. doi: 10.1111/jipb.12005 23131150

[B153] Wu G. Park M. Y. Conway S. R. Wang J.-W. Weigel D. Poethig R. S. (2009). The sequential action of miR156 and miR172 regulates developmental timing in *Arabidopsis* . Cell 138, 750–759. doi: 10.1016/j.cell.2009.06.031 19703400 PMC2732587

[B154] Xu Y. Guo C. Zhou B. Li C. Wang H. Zheng B.. (2016b). Regulation of vegetative phase change by SWI2/SNF2 chromatin remodeling ATPase BRAHMA. Plant Phys. 172, 2416–2428. doi: 10.1105/tpc.15.00854 PMC5129735 27803189

[B155] Xu M. L. Hu T. Q. Smith M. R. Poethig R. S. (2016a). Epigenetic regulation of vegetative phase change in *Arabidopsis* . Plant Cell 28, 28–41. doi: 10.1104/pp.16.01588 26704382 PMC4746683

[B156] Xu M. Leichty A. R. Hu T. Poethig R. S. (2018). *H2A.Z* promotes the transcription of *MIR156A* and *MIR156C* in *Arabidopsis* by facilitating the deposition of H3K4me3. Development 145, dev152868. doi: 10.1242/dev.152868 29361556 PMC5825843

[B157] Xu B. Yu G. Xie Z. Wen W. Zhang J. Huang B. (2019). Knockdown of *STAYGREEN* in perennial ryegrass (*Lolium perenne* l.) leads to transcriptomic alterations related to suppressed leaf senescence and improved forage quality. Plant Cell Physiol. 60, 202–212. doi: 10.1093/pcp/pcy203 30329104

[B158] Yoshikawa T. Ozawa S. Sentoku N. Itoh J.-I. Nagato Y. Yokoi S. (2013). Change of shoot architecture during juvenile-to-adult phase transition in soybean. Planta 238, 229–237. doi: 10.1007/s00425-013-1895-z 23686337

[B159] Young T. P. Augspurger C. K. (1991). Ecology and evolution of long-lived semelparous plants. Trends Ecol. Evol. 6, 285–289. doi: 10.1016/0169-5347(91)90006-J 21232483

[B160] Yu S. Lian H. Wang J.-W. (2015). Plant developmental transitions: the role of microRNAs and sugars. Curr. Opin. Plant Biol. 27, 1–7. doi: 10.1016/j.pbi.2015.05.009 26042537

[B161] Zhang S. Huang G. Zhang Y. Lv X. Wan K. Liang J.. (2022). Sustained productivity and agronomic potential of perennial rice. Nat. Sustain. doi: 10.1038/s41893-022-00997-3

[B162] Zhang X. Lin Z. Wang J. Liu H. Zhou L. Lin Z. (2019). The tin1 gene retains the function of promoting tillering in maize. Nat. Commun. 10, 5608. doi: 10.1038/s41467-019-13425-6 31811145 PMC6898233

[B163] Zhao H. Lin K. Ma L. Chen Q. Gan S. Li G. (2020). Arabidopsis NUCLEAR FACTOR y A8 inhibits the juvenile-to-adult transition by activating transcription of MIR156s. J. Exp. Bot. 71, 4890–4902. doi: 10.1093/jxb/eraa197 32445333

[B164] Zhou Y. Gan X. Viñegra de la Torre N. Neumann U. Albani M. C. (2020). Beyond flowering time: diverse roles of an APETALA2-like transcription factor in shoot architecture and perennial traits. New Phytol. 229, 444–459. doi: 10.1111/nph.16839 32745288

[B165] Zhou C.-M. Zhang T.-Q. Wang X. Yu S. Lian H. Tang H.. (2013). Molecular basis of age-dependent vernalization in cardamine flexuosa. Science 340, 1097–1100. doi: 10.1126/science.1234340 23723237

[B166] Zimmerman R. H. (1972). Juvenility and flowering in woody plants: a review. Hortic. Sci. 7, 447–455. doi: 10.21273/HORTSCI.7.5.447

