# Supplementary material for: Convergent evolution of the annual life history syndrome from perennial ancestors
Source: Front Plant Sci. 2023 Jan 4;13:1048656. doi: 10.3389/fpls.2022.1048656 (PMC9846227; doi:10.3389/fpls.2022.1048656)
Supplement: Supplementary file 1 [file DataSheet_1.zip › Supplementary material I.pdf]

Convergent evolution of the annual life history syndrome from perennial ancestors

Ane C. Hjertaas, Jill C. Preston, Kent Kainulainen, Aelys M. Humphreys and Siri Fjellheim

Supplementary material I

List of references consulted for compiling angiosperm-wide life history data (annual/perennial)

Abdallah, M. S., & De Wit, H. C. D. (1978). The Resedaceae; a taxonomical revision of the family (final instalment) Mededelingen landbouwhogeschool Wageningen No. 78-14.

Acevedo-Rodríguez, P., van Welzen, P.C., Adema, F., van der Ham, R.W.J.M. (2010). Sapindaceae. In: Kubitzki, K. (eds) Flowering Plants. Eudicots. The Families and Genera of Vascular Plants, vol 10. Springer, Berlin, Heidelberg.

African Plant Database. Conservatoire et Jardin botaniques de la Ville de Genève and South African National Biodiversity Institute, Pretoria [accessed 2018]

Al-Shehbaz, I. A., German, D. A., Mummenhoff, K., & Moazzeni, H. (2014). Systematics, tribal placements, and synopses of the Malcolmia sl segregates (Brassicaceae). Harvard Papers in Botany, 19(1), 53-71.

Albers, F., Van der Walt, J.J.A. (2007). Geraniaceae. In: Kubitzki, K. (eds) Flowering Plants · Eudicots. The Families and Genera of Vascular Plants, vol 9. Springer, Berlin, Heidelberg

Alvarado-Cárdenas, L. O., & Jiménez, J. (2015). A new species of Spigelia (Loganiaceae) from Guerrero, Mexico. Phytotaxa, 238(2), 183-189.

Amaral, M.C.E., Bittrich, V. (2014). Ochnaceae. In: Kubitzki, K. (eds) Flowering Plants. Eudicots. The Families and Genera of Vascular Plants, vol 11. Springer, Berlin, Heidelberg.

Anderberg, A.A. (2004). Primulaceae. In: Kubitzki, K. (eds) Flowering Plants · Dicotyledons. The Families and Genera of Vascular Plants, vol 6. Springer, Berlin, Heidelberg.

Anderberg, A.A. et al. (2007). Compositae. In: Kadereit, J.W., Jeffrey, C. (eds) Flowering Plants · Eudicots. The Families and Genera of Vascular Plants, vol 8. Springer, Berlin, Heidelberg.

Appel, O., Al-Shehbaz, I.A. (2003). Cruciferae. In: Kubitzki, K., Bayer, C. (eds) Flowering Plants · Dicotyledons. The Families and Genera of Vascular Plants, vol 5. Springer, Berlin, Heidelberg.

Applequist, W. L. (2005). A revision of the Malagasy endemic Talinella (Portulacaceae). Adansonia, 27(1), 47-80.

Arbo, M.M. (2007). Turneraceae. In: Kubitzki, K. (eds) Flowering Plants · Eudicots. The Families and Genera of Vascular Plants, vol 9. Springer, Berlin, Heidelberg.

Arrington, J.M., Kubitzki, K. (2003). Cistaceae. In: Kubitzki, K., Bayer, C. (eds) Flowering Plants · Dicotyledons. The Families and Genera of Vascular Plants, vol 5. Springer, Berlin, Heidelberg.

Atkins, S. (2004). Verbenaceae. In: Kadereit, J.W. (eds) Flowering Plants · Dicotyledons. The Families and Genera of Vascular Plants, vol 7. Springer, Berlin, Heidelberg.

Bailey, C. D., Al-Shehbaz, I. A., & Rajanikanth, G. (2007). Generic limits in tribe Halimolobeae and description of the new genus Exhalimolobos (Brassicaceae). Systematic Botany, 32(1), 140-156.

Ballard, H.E., de Paula-Souza, J., Wahlert, G.A. (2014). Violaceae. In: Kubitzki, K. (eds) Flowering Plants. Eudicots. The Families and Genera of Vascular Plants, vol 11. Springer, Berlin, Heidelberg.

Barboza, G.E. et al. (2016). Solanaceae. In: Kadereit, J., Bittrich, V. (eds) Flowering Plants. Eudicots. The Families and Genera of Vascular Plants, vol 14. Springer, Cham.

Barker, R. M. (1998). Notes on Zygophyllum (Zygophyllaceae) in Australia including the descriptions of five new species and one new subspecies, revised keys and typifications. Journal of the Adelaide Botanic Garden, 43-74.

Barker, W. R. (1991). A taxonomic revision of Mazus Lour.(Scrophulariaceae) in Australasia. In Papers and proceedings of the Royal Society of Tasmania (Vol. 124, No. 2, pp. 85-94).

Barker, W. R. (2017). Notes on the taxonomy of Australian Lindernia subg. Didymadenia (Linderniaceae). Swainsona, 31, 59-80.

Barker, W. R., Nesom, G. L., Beardsley, P. M., & Fraga, N. S. (2012). A taxonomic conspectus of Phrymaceae: a narrowed circumscription for Mimulus, new and resurrected genera, and new names and combinations. Phytoneuron, 39, 1-60.

Barrett, M. D., Moody, M., & Barrett, R. (2016). A review of Myriophyllum callitrichoides (Haloragaceae) in Western Australia. Telopea, 19, 207-211.

Barringer, K. (2010). A New Species of Angelonia (Plantaginaceae) From Mexico. Journal of the Botanical Research Institute of Texas, 51-54.

Bayer, C. (2003). Neuradaceae. In: Kubitzki, K., Bayer, C. (eds) Flowering Plants · Dicotyledons. The Families and Genera of Vascular Plants, vol 5. Springer, Berlin, Heidelberg.

Bayer, C., Kubitzki, K. (2003). Malvaceae. In: Kubitzki, K., Bayer, C. (eds) Flowering Plants · Dicotyledons. The Families and Genera of Vascular Plants, vol 5. Springer, Berlin, Heidelberg

Bayer, E. (1998). Alstroemeriaceae. In: Kubitzki, K. (eds) Flowering Plants · Monocotyledons. The Families and Genera of Vascular Plants, vol 3. Springer, Berlin, Heidelberg.

Bittrich, V. (1993). Caryophyllaceae. In: Kubitzki, K., Rohwer, J.G., Bittrich, V. (eds) Flowering Plants · Dicotyledons. The Families and Genera of Vascular Plants, vol 2. Springer, Berlin, Heidelberg.

Bittrich, V. (1993). Halophytaceae. In: Kubitzki, K., Rohwer, J.G., Bittrich, V. (eds) Flowering Plants · Dicotyledons. The Families and Genera of Vascular Plants, vol 2. Springer, Berlin, Heidelberg.

Bittrich, V. (2016). Lennoaceae. In: Kadereit, J., Bittrich, V. (eds) Flowering Plants. Eudicots. The Families and Genera of Vascular Plants, vol 14. Springer, Cham.

Bittrich, V., Kühn, U. (1993). Nyctaginaceae. In: Kubitzki, K., Rohwer, J.G., Bittrich, V. (eds) Flowering Plants · Dicotyledons. The Families and Genera of Vascular Plants, vol 2. Springer, Berlin, Heidelberg.

Black, J. M. (1932). Additions to the flora of South Australia No. 30. Transactions of the Royal Society of South Australia, 56, 39-47.

Bogler, D. (1998). Nolinaceae. In: Kubitzki, K. (eds) Flowering Plants · Monocotyledons. The Families and Genera of Vascular Plants, vol 3. Springer, Berlin, Heidelberg.

Bos, J.J. (1998). Dracaenaceae. In: Kubitzki, K. (eds) Flowering Plants · Monocotyledons. The Families and Genera of Vascular Plants, vol 3. Springer, Berlin, Heidelberg.

Brandbyge, J. (1993). Polygonaceae. In: Kubitzki, K., Rohwer, J.G., Bittrich, V. (eds) Flowering Plants · Dicotyledons. The Families and Genera of Vascular Plants, vol 2. Springer, Berlin, Heidelberg.

Brandegee, T.S. (1889) A collection of plants from Baja California, 1889. Proceedings of the California Academy of Sciences, Series 2, 2: 117-216

Breitwieser I., Brownsey P.J.; Nelson W.A., Wilton A.D. eds. (2010) Flora of New Zealand Online. Accessed at www.nzflora.info, 2018

Bureau of Flora and Fauna (Australia) (1996). Flora of Australia. Vol. 28, Gentianales. Canberra: Australian government publ. Service.

Cabral, E. L., Miguel, L. M., & Soto, J. D. (2012). Dos especies nuevas de Borreria (Rubiaceae) y sinopsis de las especies de Bolivia. Brittonia, 64(4), 394-412.

Calflora: Information on California plants for education, research and conservation. 2018. Berkeley, California: The Calflora Database [a non-profit organization]. Available: https://www.calflora.org/ [Accessed: 2018].

Carolin, R.C. (1993). Portulacaceae. In: Kubitzki, K., Rohwer, J.G., Bittrich, V. (eds) Flowering Plants · Dicotyledons. The Families and Genera of Vascular Plants, vol 2. Springer, Berlin, Heidelberg.

Carolin, R.C. (2007). Goodeniaceae. In: Kadereit, J.W., Jeffrey, C. (eds) Flowering Plants · Eudicots. The Families and Genera of Vascular Plants, vol 8. Springer, Berlin, Heidelberg.

Casper, S. J., & Cruz, A. J. U. (2003). Pinguicula cubensis (Lentibulariaceae)—a new insectivorous species from western Cuba (Cuba occidental). Willdenowia, 33(1), 167-172.

Cheng-Yih, W., Kubitzki, K. (1993). Circaeasteraceae. In: Kubitzki, K., Rohwer, J.G., Bittrich, V. (eds) Flowering Plants · Dicotyledons. The Families and Genera of Vascular Plants, vol 2. Springer, Berlin, Heidelberg.

Cho, S. H., Kim, B. Y., Park, H. S., Phourin, C., & Kim, Y. D. (2017). Impatiens bokorensis (Balsaminaceae), a new species from Cambodia. PhytoKeys, (77), 33.

Christenhusz, M. J., Fay, M. F., & Chase, M. W. (2017). Plants of the world. University of Chicago Press.

Christenhusz, M.J.M., Brockington, S.F., Christin, P.-A., Sage, R.F. (2014). On the disintegration of Molluginaceae: a new genus and family (Kewa, Kewaceae) segregated from Hypertelis, and placement of Macarthuria in Macarthuriaceae. Phytotaxa, 181(4), 238-242.

Cocucci, A.A. (2004). Oxalidaceae. In: Kubitzki, K. (eds) Flowering Plants · Dicotyledons. The Families and Genera of Vascular Plants, vol 6. Springer, Berlin, Heidelberg.

Conran, J.G. (1998). Anthericaceae. In: Kubitzki, K. (eds) Flowering Plants · Monocotyledons. The Families and Genera of Vascular Plants, vol 3. Springer, Berlin, Heidelberg.

Conran, J.G. (1998). Lomandraceae. In: Kubitzki, K. (eds) Flowering Plants · Monocotyledons. The Families and Genera of Vascular Plants, vol 3. Springer, Berlin, Heidelberg.

Conran, J.G., Macfarlane, T.D. (2016). Eremosynaceae. In: Kadereit, J., Bittrich, V. (eds) Flowering Plants. Eudicots. The Families and Genera of Vascular Plants, vol 14. Springer, Cham.

Cook, C.D.K. (1998). Hydrocharitaceae. In: Kubitzki, K. (eds) Flowering Plants · Monocotyledons. The Families and Genera of Vascular Plants, vol 4. Springer, Berlin, Heidelberg.

Cook, C.D.K. (1998). Pontederiaceae. In: Kubitzki, K. (eds) Flowering Plants · Monocotyledons. The Families and Genera of Vascular Plants, vol 4. Springer, Berlin, Heidelberg.

Correll, D. S., & Johnston, M. C. (1970). Manual of the vascular plants of Texas. Contributions from Texas Research Foundation. A series of botanical studies, 6.

Cross, A., Paniw, M., Scatigna, A., Kalfas, N., Anderson, B., Givnish, T., & Fleischmann, A. (2018). Systematics and evolution of small genera of carnivorous plants. In Carnivorous plants: physiology, ecology, and evolution (pp. 120-134).

Davis, P.H. (ed.) (1978). Flora of Turkey and the East Aegean islands. Vol. 6. Edinburgh

De Castro, O., & Caputo, P. (1999). A phylogenetic analysis of genus Lomelosia Rafin.(Dipsacaceae) and allied taxa. Delpinoa, 41, 29-45.

Delprete, P. G. (2014). Utricularia julianae (Lentibulariaceae), a new species from the savannas of the Oyapock River, French Guiana. Phytotaxa, 156(2), 74-78.

Devesa, J. A., & Ortega-Olivencia, A. (2003). A new species of Valantia (Rubiaceae) from Spain. Botanical Journal of the Linnean Society, 143(3), 331-335.

Diane, N., Hilger, H.H., Förther, H., Weigend, M., Luebert, F. (2016). Heliotropiaceae. In: Kadereit, J., Bittrich, V. (eds) Flowering Plants. Eudicots. The Families and Genera of Vascular Plants, vol 14. Springer, Cham.

Dillon, M. O., Leiva-González, S., & Quipuscoa-Silvestre, V. (2007). Five new species of Nolana (Solanaceae-Nolaneae) from Peru and notes on the classification of additional taxa. Arnaldoa, 14(2), 171-190.

Dong, A. Q., Zheng, X. L., Xing, F. W., & Wang, F. G. (2012). Impatiens yangshanensis (Balsaminaceae), a new species from Guangdong, China. In Annales Botanici Fennici (Vol. 49, No. 2, pp. 75-78). Finnish Zoological and Botanical Publishing Board.

Dressler, S., Repplinger, M., Bayer, C. (2014). Linaceae. In: Kubitzki, K. (eds) Flowering Plants. Eudicots. The Families and Genera of Vascular Plants, vol 11. Springer, Berlin, Heidelberg.

eFloras (2008). Published on the Internet. http://www.efloras.org [accessed 2018] Missouri Botanical Garden, St. Louis, MO & Harvard University Herbaria, Cambridge, MA

Endress, M.E., Bittrich, V. (1993). Molluginaceae. In: Kubitzki, K., Rohwer, J.G., Bittrich, V. (eds) Flowering Plants · Dicotyledons. The Families and Genera of Vascular Plants, vol 2. Springer, Berlin, Heidelberg.

Eriksen, B., Persson, C. (2007). Polygalaceae. In: Kubitzki, K. (eds) Flowering Plants · Eudicots. The Families and Genera of Vascular Plants, vol 9. Springer, Berlin, Heidelberg.

Faden, R.B. (1998). Commelinaceae. In: Kubitzki, K. (eds) Flowering Plants · Monocotyledons. The Families and Genera of Vascular Plants, vol 4. Springer, Berlin, Heidelberg.

Feuillet, C., MacDougal, J.M. (2007). Passifloraceae. In: Kubitzki, K. (eds) Flowering Plants · Eudicots. The Families and Genera of Vascular Plants, vol 9. Springer, Berlin, Heidelberg.

Fischer, E. (2004). Scrophulariaceae. In: Kadereit, J.W. (eds) Flowering Plants · Dicotyledons. The Families and Genera of Vascular Plants, vol 7. Springer, Berlin, Heidelberg.

Fischer, E., & Rahelivololona, M. E. (2016). New taxa of Impatiens (Balsaminaceae) from Madagascar VIII. Impatiens max-huberi, a new species from Marojejy and Anjanaharibe-Sud. Phytotaxa, 244(2), 191-195.

Fiz, O., Vargas, P., Alarcón, M. L., & Aldasoro, J. J. (2006). Phylogenetic relationships and evolution in Erodium (Geraniaceae) based on trnL-trnF sequences. Systematic Botany, 31(4), 739-763.

Fleischmann, A., & Rivadavia, F. (2009). Utricularia rostrata (Lentibulariaceae), a new species from the Chapada Diamantina, Brazil. Kew Bulletin, 64(1), 155-159.

Flora Mesoamericana (1994-) Published on the Internet. http://www.tropicos.org/Project/FM [accessed 2018]

Fregonezi, J. N., de Freitas, L. B., Bonatto, S. L., Semir, J., & Stehmann, J. R. (2012). Infrageneric classification of Calibrachoa (Solanaceae) based on morphological and molecular evidence. Taxon, 61(1), 120-130.

Friis, I. (1993). Urticaceae. In: Kubitzki, K., Rohwer, J.G., Bittrich, V. (eds) Flowering Plants · Dicotyledons. The Families and Genera of Vascular Plants, vol 2. Springer, Berlin, Heidelberg.

Gan, Q. L., & Li, X. W. (2016). Impatiens baokangensis (Balsaminaceae), a new species from Hubei, China. In Annales Botanici Fennici (Vol. 53, No. 3–4, pp. 145-148). Finnish Zoological and Botanical Publishing Board.

Geethakumary, M. P., Prabhukumar, K. M., Pandurangan, A. G., & Deepu, S. (2016). Exacum keralense (Gentianaceae), a new species from the Western Ghats, India. Botany Letters, 163(4), 429-433.

Gentian Research Network website, accessed 2018 [https://gentian.rutgers.edu/classABC.htm]

George, A.S. (2003). Gyrostemonaceae. In: Kubitzki, K., Bayer, C. (eds) Flowering Plants · Dicotyledons. The Families and Genera of Vascular Plants, vol 5. Springer, Berlin, Heidelberg.

Gilbert, M. G. (1987). New and interesting species of Euphorbiaceae from Ethiopia. Kew bulletin, 351-368.

Gildenhuys, E., Ellis, A. G., Carroll, S. P., & Le Roux, J. J. (2013). The ecology, biogeography, history and future of two globally important weeds: Cardiospermum halicacabum Linn. and C. grandiflorum Sw.

Gogoi, R., & Borah, S. (2015). Impatiens ashihoi, a new species of Balsaminaceae from Dibang Valley of Arunachal Pradesh, India. Phytotaxa, 238(3), 278-282.

Goldblatt, P., Manning, J.C., Rudall, P. (1998). Iridaceae. In: Kubitzki, K. (eds) Flowering Plants · Monocotyledons. The Families and Genera of Vascular Plants, vol 3. Springer, Berlin, Heidelberg.

Gottschling, M., Weigend, M., Hilger, H.H. (2016). Ehretiaceae. In: Kadereit, J., Bittrich, V. (eds) Flowering Plants. Eudicots. The Families and Genera of Vascular Plants, vol 14. Springer, Cham.

Graham, S.A. (2007). Lythraceae. In: Kubitzki, K. (eds) Flowering Plants · Eudicots. The Families and Genera of Vascular Plants, vol 9. Springer, Berlin, Heidelberg.

Grant, J. R. (2009). A Revision of Neotropical Bonyunia (Loganiaceae: Antonieae) 1. Annals of the Missouri Botanical Garden, 96(4), 541-563.

Grant, V. (1966). Genetic and taxonomic studies in Gilia. XIII. The Gilia laciniata group. Aliso: A Journal of Systematic and Floristic Botany, 6(2), 67-80.

Groeninckx, I., Janssens, S., Smets, E., & Verstraete, B. (2017). Description of 11 new Astiella (Spermacoceae, Rubiaceae) species endemic to Madagascar. European Journal of Taxonomy, 312, 1-40.

Guilliams, C. M., Veno, B. A., Simpson, M. G., & Kelley, R. B. (2013). Pectocarya anisocarpa, a new species of Boraginaceae, and a revised key for the genus in western North America. Aliso: A Journal of Systematic and Floristic Botany, 31(1), 1-13.

Gutiérrez-Báez, C., & Duno de Stefano, R. (2015). A new species of Loeselia (Polemoniaceae) from Campche, Mexico. Botanical Sciences, 93(2), 203-207.

Gutierrez, R. (2011). A phylogenetic study of the plant family Martyniaceae (Order Lamiales). Arizona State University.

Hankamp, P., Bell, C. D., & Patterson, R. (2016). Towards A Complete Species Level Phylogeny of Leptosiphon (Polemoniaceae). Madroño, 63(3), 208-219.

Hareesh, V. S., & Sabu, M. A. M. I. Y. I. L. (2017). Impatiens haridasanii (Balsaminaceae), a new species from Arunachal Pradesh, northeastern India. Phytotaxa, 326(4), 264-268.

Hart, J. M., & Henwood, M. J. (2006). A revision of Australian Trachymene (Apiaceae: Hydrocotyloideae). Australian Systematic Botany, 19(1), 11-57.

Hartmann, H.E.K. (1993). Aizoaceae. In: Kubitzki, K., Rohwer, J.G., Bittrich, V. (eds) Flowering Plants · Dicotyledons. The Families and Genera of Vascular Plants, vol 2. Springer, Berlin, Heidelberg.

Hasenstab-Lehman, K. E., & Simpson, M. G. (2012). Cat's eyes and popcorn flowers: Phylogenetic systematics of the genus Cryptantha sl (Boraginaceae). Systematic Botany, 37(3), 738-757.

Haynes, R.R., Holm-Nielsen, L.B., Les, D.H. (1998). Ruppiaceae. In: Kubitzki, K. (eds) Flowering Plants · Monocotyledons. The Families and Genera of Vascular Plants, vol 4. Springer, Berlin, Heidelberg.

Haynes, R.R., Les, D.H., Holm-Nielsen, L.B. (1998). Juncaginaceae. In: Kubitzki, K. (eds) Flowering Plants · Monocotyledons. The Families and Genera of Vascular Plants, vol 4. Springer, Berlin, Heidelberg.

Herber, B.E. (2003). Thymelaeaceae. In: Kubitzki, K., Bayer, C. (eds) Flowering Plants · Dicotyledons. The Families and Genera of Vascular Plants, vol 5. Springer, Berlin, Heidelberg.

Hilger, H.H., Weigend, M. (2016). Wellstediaceae. In: Kadereit, J., Bittrich, V. (eds) Flowering Plants. Eudicots. The Families and Genera of Vascular Plants, vol 14. Springer, Cham.

Hofmann, M., Walden, G.K., Hilger, H.H., Weigend, M. (2016). Hydrophyllaceae. In: Kadereit, J., Bittrich, V. (eds) Flowering Plants. Eudicots. The Families and Genera of Vascular Plants, vol 14. Springer, Cham.

Hofmann, U., Bittrich, V. (2016). Caprifoliaceae (with Zabelia incert. sed.). In: Kadereit, J., Bittrich, V. (eds) Flowering Plants. Eudicots. The Families and Genera of Vascular Plants, vol 14. Springer, Cham.

Hofmann, U., Bittrich, V. (2016). Morinaceae. In: Kadereit, J., Bittrich, V. (eds) Flowering Plants. Eudicots. The Families and Genera of Vascular Plants, vol 14. Springer, Cham.

Hunziker, A. T., & Subils, R. (1979). Salpiglossis, Leptoglossis and Reyesia (Solanaceae) a synoptical survey. Botanical Museum Leaflets, Harvard University, 27(1/2), 1-43.

Ihlenfeldt, HD. (2004). Pedaliaceae. In: Kadereit, J.W. (eds) Flowering Plants · Dicotyledons. The Families and Genera of Vascular Plants, vol 7. Springer, Berlin, Heidelberg.

Ihlenfeldt, HD. (2004). Trapellaceae. In: Kadereit, J.W. (eds) Flowering Plants · Dicotyledons. The Families and Genera of Vascular Plants, vol 7. Springer, Berlin, Heidelberg.

Iltis, H. H. (1956). Studies in the Capparidaceae II. The Mexican Species of Cleomella: Taxonomy and Evolution. Madroño, 13(6), 177-189.

Jepson Flora Project (eds.) 2018. Jepson eFlora, https://ucjeps.berkeley.edu/eflora/ [accessed 2018]

Jobson, R. W. (2013). Five new species of Utricularia (Lentibulariaceae) from Australia. Telopea, 15, 127-142.

Jobson, R. W., Baleeiro, P. C., & Reut, M. S. (2017). Molecular phylogeny of subgenus Polypompholyx (Utricularia; Lentibulariaceae) based on three plastid markers: diversification and proposal for a new section. Australian Systematic Botany, 30(3), 259-278.

Johansson, J. T. 2013 (and onwards). The Phylogeny of Angiosperms. Published online. http://angio.bergianska.se [accessed 2018]

Johnson, L. A., & Johnson, R. L. (2006). Morphological delimitation and molecular evidence for allopolyploidy in Collomia wilkenii (Polemoniaceae), a new species from northern Nevada. Systematic Botany, 31(2), 349-360.

Johnston, I. M. (1936). A study of the Nolanaceae. In Proceedings of the American Academy of Arts and Sciences (Vol. 71, No. 1, pp. 1-87). American Academy of Arts & Sciences.

Jonker, F. P. (1938). A monograph of the Burmanniaceae. Mededelingen van het Botanisch Museum en Herbarium van de Rijksuniversiteit te Utrecht, 51(1), 1–279.

Joyal, E. (1986). A new species of Collomia (Polemoniaceae) from the Great Basin. Brittonia, 243-248.

Kadereit, G. (2007). Menyanthaceae. In: Kadereit, J.W., Jeffrey, C. (eds) Flowering Plants · Eudicots. The Families and Genera of Vascular Plants, vol 8. Springer, Berlin, Heidelberg.

Kadereit, J.W. (1993). Papaveraceae. In: Kubitzki, K., Rohwer, J.G., Bittrich, V. (eds) Flowering Plants · Dicotyledons. The Families and Genera of Vascular Plants, vol 2. Springer, Berlin, Heidelberg.

Kalkman, C. (2004). Rosaceae. In: Kubitzki, K. (eds) Flowering Plants · Dicotyledons. The Families and Genera of Vascular Plants, vol 6. Springer, Berlin, Heidelberg.

Kamble, S. K., Forster, P. I., & Patil, B. J. (2016). Afrohybanthus indicus (Violaceae): a new species from Maharashtra, India. Phytotaxa, 252(1), 69-72.

Kers, L.E. (2003). Capparaceae. In: Kubitzki, K., Bayer, C. (eds) Flowering Plants · Dicotyledons. The Families and Genera of Vascular Plants, vol 5. Springer, Berlin, Heidelberg.

Kilian N., Hand R. & Raab-Straube E. von (general editors) 2009+ (continuously updated): Cichorieae Systematics Portal. – Published at http://cichorieae.e-taxonomy.net/portal/; accessed [2018].

Kubitzki, K. (1993). Cannabaceae. In: Kubitzki, K., Rohwer, J.G., Bittrich, V. (eds) Flowering Plants · Dicotyledons. The Families and Genera of Vascular Plants, vol 2. Springer, Berlin, Heidelberg.

Kubitzki, K. (1993). Plumbaginaceae. In: Kubitzki, K., Rohwer, J.G., Bittrich, V. (eds) Flowering Plants · Dicotyledons. The Families and Genera of Vascular Plants, vol 2. Springer, Berlin, Heidelberg.

Kubitzki, K. (2003). Droseraceae. In: Kubitzki, K., Bayer, C. (eds) Flowering Plants · Dicotyledons. The Families and Genera of Vascular Plants, vol 5. Springer, Berlin, Heidelberg.

Kubitzki, K. (2003). Resedaceae. In: Kubitzki, K., Bayer, C. (eds) Flowering Plants · Dicotyledons. The Families and Genera of Vascular Plants, vol 5. Springer, Berlin, Heidelberg.

Kubitzki, K. (2007). Haloragaceae. In: Kubitzki, K. (eds) Flowering Plants · Eudicots. The Families and Genera of Vascular Plants, vol 9. Springer, Berlin, Heidelberg.

Kubitzki, K. (2007). Malesherbiaceae. In: Kubitzki, K. (eds) Flowering Plants · Eudicots. The Families and Genera of Vascular Plants, vol 9. Springer, Berlin, Heidelberg.

Kubitzki, K., Berg, C.C. (1993). Cecropiaceae. In: Kubitzki, K., Rohwer, J.G., Bittrich, V. (eds) Flowering Plants · Dicotyledons. The Families and Genera of Vascular Plants, vol 2. Springer, Berlin, Heidelberg.

Kubitzki, K., Rudall, P.J. (1998). Asparagaceae. In: Kubitzki, K. (eds) Flowering Plants · Monocotyledons. The Families and Genera of Vascular Plants, vol 3. Springer, Berlin, Heidelberg.

Lammers, T.G. (2007). Campanulaceae. In: Kadereit, J.W., Jeffrey, C. (eds) Flowering Plants · Eudicots. The Families and Genera of Vascular Plants, vol 8. Springer, Berlin, Heidelberg.

Les, D.H. (1993). Ceratophyllaceae. In: Kubitzki, K., Rohwer, J.G., Bittrich, V. (eds) Flowering Plants · Dicotyledons. The Families and Genera of Vascular Plants, vol 2. Springer, Berlin, Heidelberg.

Lewis, D. Q. (2000). A revision of the New World species of Lindernia (Scrophulariaceae). Castanea, 93-122.

Lewis, G., Schrire, B., MacKinder, B. & Lock, M. (eds) (2005). Legumes of the world. Richmond: Royal Botanic Gardens, Kew.

Lowrie, A., & Conran, J. G. (2008). Byblis guehoi (Byblidaceae), a new species from the Kimberley, Western Australia. Telopea, 12(1), 23-29.

Luo, Q., Wang, T. J., & Zhao, L. H. (2014). Impatiens menghuochengensis sp. nov.(Balsaminaceae) from Sichuan, China. Nordic Journal of Botany, 32(6), 839-843.

Macbride, F. (1931). Spermatophytes, mostly Peruvian. III Publ. Field Mus. Nat. Hist. Chicago, Bot. Ser, 11, 13.

Manning, J. C., Goldblatt, P., & Forest, F. (2011). Molluginaceae: Adenogramma natans, A remarkable new aquatic species from Western Cape, South Africa. Bothalia - African Biodiversity and Conservation 41(1): 189-193

Manning, J., & Goldblatt, P. (2012). Plants of the greater cape floristic region. 1: the Core Cape flora. South African National Biodiversity Institute.

Mannino, A. M., Menéndez, M., Obrador, B., Sfriso, A., & Triest, L. (2015). The genus Ruppia L.(Ruppiaceae) in the Mediterranean region: an overview. Aquatic Botany, 124, 1-9.

Mansion, G. (2004). A new classification of the polyphyletic genus Centaurium Hill (Chironiinae, Gentianaceae): description of the New World endemic Zeltnera, and reinstatement of Gyrandra Griseb. and Schenkia Griseb. Taxon, 53(3), 719-740.

Mayer, V. (2016). Dipsacaceae (inclusive Triplostegia). In: Kadereit, J., Bittrich, V. (eds) Flowering Plants. Eudicots. The Families and Genera of Vascular Plants, vol 14. Springer, Cham.

Min, Y. J. (2015). Koenigia chuanzangensis (Polygonaceae), a New Species from Western Sichuan and Eastern Xizang, China. Novon: A Journal for Botanical Nomenclature, 24(3), 266-272.

Moran, R. (1977). New or renovated Polemoniaceae from Baja California, Mexico (Ipomopsis, Linanthus, Navarretia). Madroño, 24(3), 141-159.

Neubig, K. M., Blanchard Jr, O. J., Whitten, W. M., & McDaniel, S. F. (2015). Molecular phylogenetics of Kosteletzkya (Malvaceae, Hibisceae) reveals multiple independent and successive polyploid speciation events. Botanical Journal of the Linnean Society, 179(3), 421-435.

Neupane, S., Lewis, P. O., Dessein, S., Shanks, H., Paudyal, S., & Lens, F. (2017). Evolution of woody life form on tropical mountains in the tribe Spermacoceae (Rubiaceae). American Journal of Botany, 104(3), 419-438.

Ocampo, G., & Columbus, J. T. (2012). Molecular phylogenetics, historical biogeography, and chromosome number evolution of Portulaca (Portulacaceae). Molecular phylogenetics and evolution, 63(1), 97-112.

Ohsako, T., Yamane, K., & Ohnishi, O. (2002). Two new Fagopyrum (Polygonaceae) species, F. gracilipedoides and F. jinshaense from Yunnan, China. Genes & genetic systems, 77(6), 399-408.

Otero, A., Jiménez-Mejías, P., Valcárcel, V., & Vargas, P. (2014). Molecular phylogenetics and morphology support two new genera (Memoremea and Nihon) of Boraginaceae ss. Phytotaxa, 173(4), 241-277.

Oxelman, B., Kornhall, P., Norman, E.M. (2004). Buddlejaceae. In: Kadereit, J.W. (eds) Flowering Plants · Dicotyledons. The Families and Genera of Vascular Plants, vol 7. Springer, Berlin, Heidelberg.

PlantNET (The NSW Plant Information Network System). Royal Botanic Gardens and Domain Trust, Sydney. https://plantnet.rbgsyd.nsw.gov.au [accessed 2018]

Porter, D. M. (1969). The genus Kallstroemia (Zygophyllaceae). Contributions from the Gray Herbarium of Harvard University, (198), 41-153.

Prasad, K. S., & Raveendran, K. (2012). Canscora bhatiana (Gentianaceae), a new species from Kerala, India. International Journal of Plant, Animal and Environmental Sciences, 2(4), 197-201.

Raju, A. S., & Radhakrishna, J. (2018). Pollination ecology of the annual herb, Hedyotis brachiata (Rubiaceae). Annali di Botanica, 8, 9-16.

Rechinger, K.H. & Fischer, M.A. (eds) (1981). Flora Iranica: Flora des iranischen Hochlandes und der umrahmenden Gebirge, Persien, Afghanistan, Teile von West-Pakistan, Nord-Iraq, Azerbaidjan, Turkmenistan. Lief. 147, Scrophulariaceae, 1. Graz

Rohwer, J.G., Berg, C.C. (1993). Moraceae. In: Kubitzki, K., Rohwer, J.G., Bittrich, V. (eds) Flowering Plants · Dicotyledons. The Families and Genera of Vascular Plants, vol 2. Springer, Berlin, Heidelberg.

Rydberg, P. A. (1901). Studies on the Rocky Mountain Flora.-IV. Bulletin of the torrey Botanical Club, 28(1), 20-38.

Schaefer, H., Renner, S.S. (2010). Cucurbitaceae. In: Kubitzki, K. (eds) Flowering Plants. Eudicots. The Families and Genera of Vascular Plants, vol 10. Springer, Berlin, Heidelberg.

Schneider, E.L., Williamson, P.S. (1993). Nymphaeaceae. In: Kubitzki, K., Rohwer, J.G., Bittrich, V. (eds) Flowering Plants · Dicotyledons. The Families and Genera of Vascular Plants, vol 2. Springer, Berlin, Heidelberg.

Schwarzbach, A.E. (2004). Plantaginaceae. In: Kadereit, J.W. (eds) Flowering Plants · Dicotyledons. The Families and Genera of Vascular Plants, vol 7. Springer, Berlin, Heidelberg.

Schäferhoff, B., Müller, K. F., & Borsch, T. (2009). Caryophyllales phylogenetics: disentangling Phytolaccaceae and Molluginaceae and description of Microteaceae as a new isolated family. Willdenowia, 209-228.

Shao, J. R., Zhou, M. L., Zhu, X. M., Wang, D. Z., & Bai, D. Q. (2011). Fagopyrum wenchuanense and Fagopyrum qiangcai, two new species of Polygonaceae from Sichuan, China. Novon: A Journal for Botanical Nomenclature, 21(2), 256-261.

Sheahan, M.C. (2007). Zygophyllaceae. In: Kubitzki, K. (eds) Flowering Plants · Eudicots. The Families and Genera of Vascular Plants, vol 9. Springer, Berlin, Heidelberg.

Sheahan, M.C. (2010). Nitrariaceae. In: Kubitzki, K. (eds) Flowering Plants. Eudicots. The Families and Genera of Vascular Plants, vol 10. Springer, Berlin, Heidelberg.

Sheahan, M.C. (2010). Tetradiclidaceae. In: Kubitzki, K. (eds) Flowering Plants. Eudicots. The Families and Genera of Vascular Plants, vol 10. Springer, Berlin, Heidelberg.

Smith, G.F., Van Wyk, BE. (1998). Asphodelaceae. In: Kubitzki, K. (eds) Flowering Plants · Monocotyledons. The Families and Genera of Vascular Plants, vol 3. Springer, Berlin, Heidelberg.

Soltis, D.E. (2007). Saxifragaceae. In: Kubitzki, K. (eds) Flowering Plants · Eudicots. The Families and Genera of Vascular Plants, vol 9. Springer, Berlin, Heidelberg.

Soza, V. L., & Olmstead, R. G. (2010). Evolution of breeding systems and fruits in New World Galium and relatives (Rubiaceae). American Journal of Botany, 97(10), 1630-1646.

Sparre, B., & Andersson, L. (1991). Taxonomic revision of the Tropaeolaceae. Opera Botanica 108: 1-139.

Speta, F. (1998). Hyacinthaceae. In: Kubitzki, K. (eds) Flowering Plants · Monocotyledons. The Families and Genera of Vascular Plants, vol 3. Springer, Berlin, Heidelberg.

Steiner, K. E. (2006). Two new species of Nemesia (Scrophulariaceae) from southern Africa. Bothalia, 36(1), 39-44.

Steiner, K. E. (2009). Three new species of Diascia (Scrophulariaceae) from the Western Cape, South Africa. Bothalia, 39(1), 11-17.

Steiner, K. E. (2009). Two new species of Nemesia (Scrophulariaceae) from arid areas of the Northern Cape, South Africa. Bothalia, 39(1), 67-72.

Steiner, K. E. (2010). Two new species of Nemesia (Scrophulariaceae) from the southern Cape, South Africa. Bothalia, 40(2), 161-166.

Steiner, K. E. (2011). A new endemic Diascia (Scrophulariaceae) threatened by proposed tungsten mining in the Western Cape. South African Journal of Botany, 77(3), 777-781.

Stevens, P.F. (2007). Hypericaceae. In: Kubitzki, K. (eds) Flowering Plants · Eudicots. The Families and Genera of Vascular Plants, vol 9. Springer, Berlin, Heidelberg.

Ståhl, B. (2004). Theophrastaceae. In: Kubitzki, K. (eds) Flowering Plants · Dicotyledons. The Families and Genera of Vascular Plants, vol 6. Springer, Berlin, Heidelberg.

Ståhl, B., Anderberg, A.A. (2004). Maesaceae. In: Kubitzki, K. (eds) Flowering Plants · Dicotyledons. The Families and Genera of Vascular Plants, vol 6. Springer, Berlin, Heidelberg.

Ståhl, B., Anderberg, A.A. (2004). Myrsinaceae. In: Kubitzki, K. (eds) Flowering Plants · Dicotyledons. The Families and Genera of Vascular Plants, vol 6. Springer, Berlin, Heidelberg.

Sukhorukov, A. P., & Kushunina, M. (2016). Taxonomic revision and distribution of herbaceous Paramollugo (Molluginaceae) in the Eastern Hemisphere. PhytoKeys, (73), 93.

Sutthisaksopon, P., Chantaranothai, P., & Simpson, D. A. (2014). A new species of the genus Vandellia (Linderniaceae) from Thailand. Phytotaxa, 167(1), 127-132.

Tamura, M. (1993). Ranunculaceae. In: Kubitzki, K., Rohwer, J.G., Bittrich, V. (eds) Flowering Plants · Dicotyledons. The Families and Genera of Vascular Plants, vol 2. Springer, Berlin, Heidelberg.

Tate, J. A., & Simpson, B. B. (2003). Paraphyly of Tarasa (Malvaceae) and diverse origins of the polyploid species. Systematic Botany, 28(4), 723-737.

Taylor, C. M. (1994). Revision of Tetragonia (Aizoaceae) in South America. Systematic Botany, 575-589.

Terrell, E. E. (1996). Revision of Houstonia (Rubiaceae-Hedyotideae). Systematic Botany Monographs, 1-118.

Theisen, I., Fischer, E. (2004). Myoporaceae. In: Kadereit, J.W. (eds) Flowering Plants · Dicotyledons. The Families and Genera of Vascular Plants, vol 7. Springer, Berlin, Heidelberg.

Thiede, J., Eggli, U. (2007). Crassulaceae. In: Kubitzki, K. (eds) Flowering Plants · Eudicots. The Families and Genera of Vascular Plants, vol 9. Springer, Berlin, Heidelberg.

Thiv, M., & Kadereit, J. W. (2002). A morphological cladistic analysis of Gentianaceae-Canscorinae and the evolution of anisomorphic androecia in the subtribe. Systematic Botany, 780-788.

Todzia, C.A. (1993). Ulmaceae. In: Kubitzki, K., Rohwer, J.G., Bittrich, V. (eds) Flowering Plants · Dicotyledons. The Families and Genera of Vascular Plants, vol 2. Springer, Berlin, Heidelberg.

Ulbrich, E. (1908). Malvaceae austro-americanae imprimis andinae. In: Urban, I. (ed) Plantae novae andinae imprimis Weberbauerianae. Part IV. Botanische Jahrbücher fur Systematik, Pflanzengeschichte und Pflanzengeographie 42: 49-177.

Ulbrich, E. (1916). Malvaceae andinae novae vel criticae imprimis Weberbauerianae. In: Gilg, E. (ed) Plantae novae andinae imprimis imprimis Weberbauerianae. Part VII. Beiblatt zu den Botanische Jahrbücher 117. 80 pp.

Umemoto, H., Yokota, M., & Kokubugata, G. (2015). Reconsideration for Occurrence of Mazus goodenifolius (Phrymaceae) in Miyazaki Prefecture, Japan using Molecular and Morphological Data. Bulletin of the National Museum of Nature and Science. Series B, 41, 61-67.

Urban, I., Gilg, E. Monographia Loasacearum. Nova Acta Caes. Leop.-Carol. German. Natl. Cur., 76 (1900), pp. 1-368

Ventenat, E. (1804). Josephinia imperatricis. Jardin de la Malmaison 2: t. 67. Crapelet, Paris

Verhoek, S. (1998). Agavaceae. In: Kubitzki, K. (eds) Flowering Plants · Monocotyledons. The Families and Genera of Vascular Plants, vol 3. Springer, Berlin, Heidelberg.

VicFlora (2018). Flora of Victoria, Royal Botanic Gardens Victoria. Available online: https://vicflora.rbg.vic.gov.au [accessed 2018]

von Mering, S., Kadereit, J. W., Seberg, O., Petersen, G., Barfod, A. S., & Davis, J. I. (2010). Phylogeny, systematics and recircumscription of Juncaginaceae–a cosmopolitan wetland family. Diversity, phylogeny, and evolution in the monocotyledons, 55-79.

Wagstaff, S.J. (2004). Tetrachondraceae. In: Kadereit, J.W. (eds) Flowering Plants · Dicotyledons. The Families and Genera of Vascular Plants, vol 7. Springer, Berlin, Heidelberg.

Wannan, B. S. (2016). Three new species in Lindernia All. sl (Linderniaceae) for Australia. Austrobaileya, 508-523.

Waterfall, U. T. (1967). Physalis in Mexico, Central America and the West Indies. Rhodora, 69(777), 82-120.

Watson, D. R. A. (2013). Datura arenicola (Solanaceae): A New Species in the New Section Discola from Baja California Sur, Mexico. Madroño, 60(3), 217-228.

Watson, S. (1888). Contributions to American botany. In Proceedings of the American Academy of Arts and Sciences (Vol. 24, pp. 36-87). American Academy of Arts & Sciences.

Weber, A. (2004). Gesneriaceae. In: Kadereit, J.W. (eds) Flowering Plants · Dicotyledons. The Families and Genera of Vascular Plants, vol 7. Springer, Berlin, Heidelberg.

Weberling, F., Bittrich, V. (2016). Valerianaceae. In: Kadereit, J., Bittrich, V. (eds) Flowering Plants. Eudicots. The Families and Genera of Vascular Plants, vol 14. Springer, Cham.

Webster, G.L. (2014). Euphorbiaceae. In: Kubitzki, K. (eds) Flowering Plants. Eudicots. The Families and Genera of Vascular Plants, vol 11. Springer, Berlin, Heidelberg.

Weese, T. L., & Johnson, L. A. (2001). Saltugilia latimeri: a new species of Polemoniaceae. Madrono, 198-204.

Weigend, M. (2004). Loasaceae. In: Kubitzki, K. (eds) Flowering Plants · Dicotyledons. The Families and Genera of Vascular Plants, vol 6. Springer, Berlin, Heidelberg.

Weigend, M. (2007). Ledocarpaceae. In: Kubitzki, K. (eds) Flowering Plants · Eudicots. The Families and Genera of Vascular Plants, vol 9. Springer, Berlin, Heidelberg.

Weigend, M., Selvi, F., Thomas, D.C., Hilger, H.H. (2016). Boraginaceae. In: Kadereit, J., Bittrich, V. (eds) Flowering Plants. Eudicots. The Families and Genera of Vascular Plants, vol 14. Springer, Cham.

Welman, W. G. (2000) FSA contributions 16: Sphenocleaceae. Bothalia 30(1): 31-33.

Western Australian Herbarium (1998–). Florabase—the Western Australian Flora. Department of Biodiversity, Conservation and Attractions. https://florabase.dpaw.wa.gov.au/ [accessed 2018]

Whitfoord, C. (2012) 134. Ochnaceae. Flora Mesoamericana, Vol. 3(2), Ochnaceae, pp. 26

Wilbur, R. L. (1955). A revision of the North American genus Sabatia (Gentianaceae). Rhodora, 57(673), 1-33.

Wilbur, R. L. (1984). A synopsis of the genus Halenia (Gentianaceae) in Mexico. Rhodora, 311-337.

Wilken, D.H. (2004). Polemoniaceae. In: Kubitzki, K. (eds) Flowering Plants · Dicotyledons. The Families and Genera of Vascular Plants, vol 6. Springer, Berlin, Heidelberg.

Wilkinson, H.P., Wanntorp, L. (2007). Gunneraceae. In: Kubitzki, K. (eds) Flowering Plants · Eudicots. The Families and Genera of Vascular Plants, vol 9. Springer, Berlin, Heidelberg.

Wilson, P. (1928). Two new species of Portulaca from Mexico. Torreya, 28-29.

Witham, C. W., & Zika, P. F. (2008). Juncus digitatus (Juncaceae), A new annual rush from Shasta County, California, USA. Journal of the Botanical Research Institute of Texas, 775-781.

Wohlhauser, S., & Callmander, M. W. (2012). Exacum alberti-grimaldii Wohlh. & Callm.(Gentianaceae), a new species endemic to northern Madagascar. Candollea, 67(2), 373-378.

Worley, A. C., Ghazvini, H., & Schemske, D. W. (2009). A phylogeny of the genus Polemonium based on amplified fragment length polymorphism (AFLP) markers. Systematic botany, 34(1), 149-161.

Yadav, S. R., & Janarthanam, M. K. (1994). Hydatellaceae: a new family to Indian flora with a new species. Rheedea 4(1): 17-20.
