# Supplementary material for: Convergent evolution of the annual life history syndrome from perennial ancestors
Source: Front Plant Sci. 2023 Jan 4;13:1048656. doi: 10.3389/fpls.2022.1048656 (PMC9846227; doi:10.3389/fpls.2022.1048656)
Supplement: Supplementary file 1 [file DataSheet_1.zip › Supplementary material II.pdf]

Convergent evolution of the annual life history syndrome from perennial ancestors

Ane C. Hjertaas, Jill C. Preston, Kent Kainulainen, Aelys M. Humphreys and Siri Fjellheim

Supplementary material II

List of experts consulted for compiling angiosperm-wide life history data (annual/perennial)

**Expert consulted**	**Family**
Erin Tripp	Acanthaceae
Arne Anderberg	Asteraceae
Bruce Baldwin	Asteraceae
Mark W. Bierner	Asteraceae
Luc Brouillet	Asteraceae
Lal Babu Chaudhary	Asteraceae
Zita Ferreira	Asteraceae
Mercè Galbany-Casals	Asteraceae
Ronald L. Hartman	Asteraceae
Huseyin Inceer	Asteraceae
Joachim Kadereit	Asteraceae
Martha Kandziora	Asteraceae
Per-Ola Karis	Asteraceae
John C. Manning	Asteraceae
Evgeny Mavrodiev	Asteraceae
Iraj Mehregan	Asteraceae
José A. Mejías	Asteraceae
Abigail Moore	Asteraceae
Christoph Oberprieler	Asteraceae
Santiago Ortiz	Asteraceae
Pieter Pelser	Asteraceae
Pimwadee Pornpongrungrueng	Asteraceae
Mike Powell	Asteraceae
Edward E. Schilling	Asteraceae

Angelo Schneider	Asteraceae
John C. Semple	Asteraceae
Marek Slovák	Asteraceae
Akiko Soejima	Asteraceae
Tod Stuessy	Asteraceae
Joan Vallès	Asteraceae
Kuniaki Watanabe	Asteraceae
Mark Hughes	Begoniaceae
Catherine Kidner	Begoniaceae
Peter Moonlight	Begoniaceae
Pamela Puppo	Calceolariaceae
Raul Pozner	Calyceraceae
Bill Barker	Celastraceae
Max Weigend	Loasaceae
Frank Almeda	Melastomataceae
Magnus Lidén	Papaveraceae
John MacDougal	Passifloraceae
Guido Mathieu	Piperaceae
Marinne Le Roux	Santalaceae
