# Supplementary material for: Convergent evolution of the annual life history syndrome from perennial ancestors
Source: Front Plant Sci. 2023 Jan 4;13:1048656. doi: 10.3389/fpls.2022.1048656 (PMC9846227; doi:10.3389/fpls.2022.1048656)
Supplement: Supplementary file 1 [file DataSheet_1.zip › Supplementary material III.pdf]

Convergent evolution of the annual life history syndrome from perennial ancestors

Ane C. Hjertaas, Jill C. Preston, Kent Kainulainen, Aelys M. Humphreys and Siri Fjellheim

Supplementary material III

Estimating phylogenetic signal in the distribution of annuals among angiosperm families

To estimate the phylogenetic signal in how annuals are distributed among angiosperm families we used three commonly used measures, Pagel's λ, Blomberg's K and the D-statistic (Pagel 1997, Pagel 1999, Blomberg, Garland et al. 2003, Fritz and Purvis 2010), and a phylogenetic tree with family-level resolution that includes 358 angiosperm families (Fig. 1, main article; Magallón, Gomez-Acevedo et al. 2015). The subjective nature of taxonomic categories makes summarising life history variation by family somewhat artificial; therefore, the data were analysed both as proportions and numbers: Pagel's λ and Blomberg's K were used to analyse the proportion annual species per family and the D-statistic for treating life history variation as a binary variable (presence/absence of annual species in a family). We used both λ and K because they treat the data (e.g. tip branches) slightly differently, and their performance is known to differ under some circumstances (Münkemüller, Lavergne et al. 2012). Pagel's λ was calculated using the 'transformPhylo.ML' function in the R (R Development Core Team 2014) package motmot (Thomas and Freckleton 2012), Blomberg's K using 'phylosig' in phytools (Revell 2012) and the D-statistic using 'phylo.d' in caper (Orme, Freckleton et al. 2014). The proportions data were normalised by logit transformation prior to analysis. Significance of K and D were determined using 1,000 randomizations each.

We repeated the calculations with annuals differently coded depending on the definition (footnote 1). All tests provided support for some phylogenetic signal in how annuals are distributed among angiosperm families. For the 119 families with obligate annuals (both terrestrial and aquatic), $\lambda = 0.34$ ($\Delta AICc \geq 9.8$ compared to both a phylogenetically random distribution (equivalent to $\lambda = 0$) and Brownian motion (equivalent to $\lambda = 1$, where differences among the families are proportional to the phylogenetic distances among them); $K = 0.72$, which is significantly different from a phylogenetically random distribution ($P = 0.003$); and $D = 0.41$, which is significantly different from a random distribution ($P=0$) but not from Brownian motion ($P=0.06$). The results are qualitatively the same for the 128 families that also include facultative annuals and biennial species:

$\lambda = 0.37$ (ΔAICc $\geq$ 10.8 compared to both a random and Brownian distribution); Blomberg's K = 0.72 (P=0.003 compared to a random distribution); and D = 0.42 (significantly different from a random distribution but not Brownian motion (P=0 and 0.08, respectively). Thus, annuals are present in all major clades of angiosperms and their distribution among families is strongly phylogenetically structured.

References - Appendix III

Blomberg, S. P., T. Garland and A. R. Ives (2003). "Testing for phylogenetic signal in comparative data: behavioural traits are more labile." Evolution **57**(4): 717-745.

Fritz, S. A. and A. Purvis (2010). "Selectivity in Mammalian Extinction Risk and Threat Types: a New Measure of Phylogenetic Signal Strength in Binary Traits." Conservation Biology **24**(4): 1042-1051.

Magallón, S., S. Gomez-Acevedo, L. L. Sanchez-Reyes and T. Hernandez-Hernandez (2015). "A metacalibrated time-tree documents the early rise of flowering plant phylogenetic diversity." New Phytologist **207**(2): 437-453.

Münkemüller, T., S. Lavergne, B. Bzeznik, S. Dray, T. Jombart, K. Schiffers and W. Thuiller (2012). "How to measure and test phylogenetic signal." Methods in Ecology and Evolution **3**(4): 743-756.

Orme, C. D. L., R. P. Freckleton, G. Thomas, T. Petzoldt, S. A. Fritz and N. Isaac (2014). caper: Comparable Analyses of Phylogenetics and Evolution in R**:** R package version 0.4.

Pagel, M. (1997). "Inferring evolutionary processes from phylogenies." Zoologica Scripta **26**(4): 331-348.

Pagel, M. (1999). "Inferring the historical patterns of biological evolution." Nature **401**(6756): 877-884.

R Development Core Team. (2014). "R: A language and environment for statistical computing." from://www.R-project.org.

Revell, L. J. (2012). "Phytools: An R package for phylogenetic comparative biology (and other things)." Methods in Ecology and Evolution **3**: 217-223.

Thomas, G. H. and R. P. Freckleton (2012). "MOTMOT: models of trait macroevolution on trees." Methods in Ecology and Evolution **3**: 145-151.
